# Hyperphosphorylated tau mediates neuronal death by inducing necroptosis and inflammation in Alzheimer’s disease

**DOI:** 10.1186/s12974-022-02567-y

**Published:** 2022-08-15

**Authors:** Yue Dong, Hanqiao Yu, Xueqi Li, Kelong Bian, Yayuan Zheng, Mingrui Dai, Xuejian Feng, Yao Sun, Yu He, Bin Yu, Haihong Zhang, Jiaxin Wu, Xianghui Yu, Hui Wu, Wei Kong

**Affiliations:** 1 National Engineering Laboratory for AIDS Vaccine, School of Life Sciences, Jilin University, Changchun, 130012 People’s Republic of China; 2 Key Laboratory for Molecular Enzymology and Engineering, The Ministry of Education, School of Life Sciences, Jilin University, Changchun, 130012 People’s Republic of China

**Keywords:** Hyperphosphorylated tau, Neuronal death, Necroptosis, Inflammation, NF-κB, Alzheimer’s disease

## Abstract

**Background:**

Progressive neuronal death is the key pathological feature of Alzheimer’s disease (AD). However, the molecular mechanisms underlying the neuronal death in AD patients have not been fully elucidated. Necroptosis reportedly activates and induces neuronal death in patients with Alzheimer’s disease (AD); however, the main mediators and mechanisms underlying necroptosis induction in AD remain elusive.

**Methods:**

The function of hyperphosphorylated tau (pTau) in inducing necroptosis in neuronal cell was examined using Western blotting, RT-PCR and flow cytometry. Tau-induced inflammation was identified via RNA sequencing and transwell assay. Pharmacological methods and CRISPR–Cas9 technology were used to verify the role of necrosome proteins in pTau-stimulated neuronal death and inflammation. TauP301S model mice were treated with Nec-1 s to evaluate the role of necroptosis in tau pathology.

**Results:**

Hyperphosphorylated tau could induce necroptosis in neuronal cells by promoting the formation of the RIPK1/RIPK3/MLKL necrosome. In addition, pTau significantly stimulated cell-autonomous overexpression of cytokines and chemokines via the intracellular nuclear factor kappa B (NF-κB) signaling pathway. Importantly, the RIPK1/RIPK3/MLKL axis was essential for the pTau-mediated NF-κB activation and cytokine storm. Furthermore, necroptosis stimulation, NF-κB activation, and cytokine induction have been detected in TauP301S mice and blocking necroptosis markedly ameliorated behavioral defects and excessive neuroinflammation in AD mice.

**Conclusions:**

Our study, for the first time, revealed that pTau contributes to neuronal death by inducing necroptosis and inflammation, mediated by activating the RIPK1/RIPK3/MLKL and NF-κB pathways, thereby delineating the hierarchical molecular network of neuronal necroptosis induction in AD.

**Supplementary Information:**

The online version contains supplementary material available at 10.1186/s12974-022-02567-y.

## Introduction

Alzheimer’s disease (AD) is a devastating neurodegenerative disorder that is the most common cause of dementia. Apart from deposited amyloid-β (Aβ) plaques and accumulated hyperphosphorylated tau (pTau), severe progressive neuronal death is another key pathological feature of AD [1–3]. However, the precise mechanisms underlying neuronal death in AD remain to be comprehensively elucidated.

Necroptosis is a form of programmed necrotic cell death that is induced by the activation of death receptors (DRs) including tumor necrosis factor (TNF) receptor 1 (TNFR1) [4, 5]. When necroptosis occurs, activated RIPK1 recruits RIPK3 to trigger its phosphorylation [6, 7]. Activated RIPK3 then phosphorylates the mixed lineage kinase domain-like protein (MLKL), leading to the assembly of the necrosome [8]. Phosphorylated MLKL further forms an oligomer and translocates to the plasma membrane, causing cell death by disrupting the cell membrane [9, 10]. Caccamo et al. have revealed that RIPK1 levels were upregulated in human AD brains and that MLKL phosphorylation and oligomerization were also increased, indicating that necroptosis is activated in AD brains [11]. Nevertheless, there has been no further undertaking to date regarding the key trigger and related mechanism for necroptosis activation in AD brains.

Tau protein is a major microtubule-associated protein (MAPT) in the mammalian nervous system, while abnormal hyperphosphorylation of tau has been identified in AD and related tauopathies [12, 13]. pTau, at several residues including Ser393/404, Ser202 and Thr 205, accumulates intraneuronally to form NFTs [14]. As a result, communication between neuronal malfunctions and neuronal death occurs. Although existing data firmly support the key role of Aβ aggregates in mediating AD, multiple lines of evidence suggest that accumulated pTau may be the primary contributor to neurodegeneration during AD [15]. Clinicopathologic correlation studies conducted by Braak et al. have demonstrated that the accumulation of NFTs correlates well with the status of cognitive impairment during the course of AD, which is referred to as “Braak stages” [16]. Subsequently, a large body of neuropathologic analyses has strongly supported that cognitive deficit and neuronal loss in AD are primarily driven by tau pathology and not Aβ deposition [17, 18]. Caccamo et al. have reported that all three necrosome protein levels failed to predict the Aβ burden in human AD brains. In contrast, the increase in both RIPK1 and MLKL significantly correlated with the Braak stage, indicating that abnormally pTau, but not aggregated Aβ, might be a pivotal trigger for neuronal necroptosis activation in AD brains. To date, direct evidence implicating the role of tau phosphorylation in neuronal necroptosis, as well as relevant downstream events, remains elusive.

Apart from Aβ plaques and NFTs, neuroinflammation also plays an important role in the progression of AD [19]. Microglial activation and subsequent release of pro-inflammatory factors play a significant role in neuronal damage [20]. Following phagocytosis of Aβ by microglia, Aβ activates the NLRP3 inflammasome, resulting in caspase-1 activation and interleukin (IL)-1β maturation and release [21]. In the TauP301S tauopathy mouse model, microglial inactivation attenuates neuroinflammation and tau-mediated neurodegeneration, indicating that microglia contribute to neuronal death in models of tau-mediated neurodegeneration [22, 23]. Furthermore, elevated levels of pro-inflammatory cytokines, including TNF-α and IL-6, were detected in the serum and brain of patients with AD when compared with controls [24, 25]. In addition to pro-inflammatory cytokines, chemokines are also important contributors to neuroinflammation, which reportedly increase in the brain and induce microglial chemotaxis. Thus, neuroinflammation in AD reflects an abnormally excessive activation of microglia, overexpression of pro-inflammatory factors, and neuronal death. In addition, activated RIPK1 has been shown to promote neuroinflammation during the pathogenesis of several neurodegenerative disorders [26–28]. Thus, these findings suggested that RIPK1 is a critical regulator of cell death and inflammation in neurodegenerative diseases.

To precisely identify the initiator, mechanism, and downstream events of necroptosis activation in AD, we investigated the role of pTau in neuronal necroptosis and aimed to clarify relevant mechanisms. Our data revealed that pTau could induce necroptosis in neuronal cells. Furthermore, tau pathology can promote cell-autonomous expression of pro-inflammatory cytokines and chemokines by stimulating the NF-κB signaling pathway. Notably, the RIPK1–RIPK3–MLKL axis is responsible for NF-κB activation and further cytokine storm driven by neurotoxic tau. We also detected necroptosis stimulation, NF-κB activation, and induced cytokine storms in a the TauP301S AD mouse model and observed that RIPK1 inhibition effectively ameliorated the behavioral deficits and excessive neuroinflammation activation in AD mice. Thus, our study, for the first time, demonstrated that pTau functions as a key trigger for neuronal death by inducing necroptosis and neuroinflammation.

## Materials and methods

### Cell culture and transfection

HEK 293 T and HT22 was purchased from the American Type Culture Collection. HEK 293 T and HT22 were cultured in Dulbecco’s modified Eagle’s medium (12100061, Invitrogen) with 10% fetal bovine serum (A3160901, Gibco) and 100 U/ml penicillin–streptomycin and incubated at 37 °C under 5% CO_2_. Transfections of cells were performed using Hieff Trans™ Liposomal Transfection Reagent (40802ES03, Yeasen Biotechnology) according to the manufacturer’s instructions.

### Reagents and antibodies

The following commercial antibodies and reagents were used: Necrostatin-1 (S8037, Selleck); Necrostatin 1S (S8641, Selleck); Z-VAD-FMK (S0723, Selleck); TPCA-1(S2824, Selleck); PH-797804 (S2726, Selleck); SP600125 (S1460, Selleck); C-176 (S6575, Selleck); QNZ (S4902, Selleck); RIPK1 (1:1000, 551041, BD Biosciences); RIPK3 (1:1000, NBP1-77299, NOVUS); MLKL (1:2000, AP14272B, Abcepta); pMLKL (1:1000, 37333S, Cell Signaling Technology); Flag (1:1000, 20543-1-AP, Proteintech); caspase3 (1:1000, 19677-1-AP, Proteintech); HMGB1 (1:1000, 10829-1-AP, Proteintech); p65 (1:1000, 66535-1-Ig, Proteintech); GAPDH (1:10,000, 60004-1-Ig, Proteintech); Histone H3 (1:1000, 17168-1-AP, Proteintech);IL6 (1:1000, 66146-1-Ig, Proteintech); CD68 (1:1000, 28058-1-AP, Proteintech); TMEM119 (1:1000, 27585-1-AP, Proteintech); p-p65 (1:200, sc-136548, Santa Cruz); IKK (1:500, AF6009, Affinity); p-IKK (1:500, AF3013, Affinity); IKbα (1:1000, 10268-1-AP, Proteintech); p-IKbα (1:500, AF2002, Affinity); Ccl5 (1:500, AF5151, Affinity); IFNb1 (1:500, DF6471, Affinity); pTau396 (1:1000, 829,001, Biolegend); AT8 (1:1000, MN1020, Thermo); Goat Anti-Mouse-HRP IgG (1:10,000, 115-035-003, Jackson ImmunoResearch); Goat Anti-Rabbit-TRITC IgG (1:200, 111-025-045, Jackson ImmunoResearch); Goat Anti-Rabbit-HRP IgG (1:10,000, 111-005-003, Jackson ImmunoResearch).

### Plasmid construction

HTau (0N4R) was obtained from GenScript. Flag tag sequence was added to the N terminus of Tau by PCR. Then the tagged Tau was inserted into VR1012 vector for mammalian expression. P301S Tau mutant was generated using site-direct mutagenesis. The VR1012 plasmid was described previously [34].

### Western blotting and immunoprecipitation

Cells were trypsinized and collected by centrifugation. After 2 × wash with PBS, cells were lysed with RIPA buffer (Beyotime) supplemented with 1 mM PMSF, 1 × EDTA-free protease inhibitor mixture (Roche), 1 × phosphatase inhibitor (Beyotime) and incubated on ice for 30 min. Protein samples were mixed with 4 × SDS loading buffer and were boiled for 10 min at 100 °C. Protein samples were load into a 10% or a 12% polyacrylamide gel for separation by SDS–PAGE and then transferred to PVDF membranes. The membranes were blocked with 5% nonfat milk, and then they were incubated with primary antibodies at 4℃ overnight. The membranes were incubated with secondary antibodies for 1 h at room temperature and then incubated with ECL luminescence reagent (Meilun Biotechnology) for 1 min and then exposed using Tanon 5200 Chemiluminescence Imaging System (Tanon).

For immunoprecipitation, cells were lysed with NP-40 buffer supplemented with 1 mM PMSF, 1 × EDTA-free protease inhibitor mixture (Roche), 1 × phosphatase inhibitor (Beyotime) at 4 °C for 1 h. The lysate was incubated FLAG-tagged beads (Sigma, A2220) for 4 h, or overnight with RIPK1 antibody followed by 3 h incubation with Protein G-Agraose (Roche, 11719416001). Beads were washed six times and boiled in SDS loading buffer for 10 min. For image acquisition and analysis, ImageJ was used. All primary images of Western blots were presented in Additional file 9.

### Cell death assays

Cells were plated in a 24- or 96-well plate overnight. Then cells were transfected with VR1012 or VR1012-TauP301S. The transfection medium was replaced after 4 ~ 6 h and then were treated with compounds at indicated concentrations. After 24 h or 48 h of transfection, cell death was measured using lactate dehydrogenase (LDH) release kit (Beyotime) according to the manufacturer’s instructions. The percentage of cell death per well was calculated by comparing to that of the maximal cell death with LDH Release Reagent after deducting background signal in non-induced cells. The absorbance was measured using an ELx800 universal microplate reader (BIO-TEK).

### Flow cytometry

Cell death was analyzed using flow cytometry according to the instructions of Annexin V/PI staining kit (KeyGen Biotechology). Briefly, after 48 h of transfection, cells were collected and resuspended in 500 μl binding buffer containing 5 μl FITC-annexin V and 5 μl propidium iodide (PI) for 15 min in the dark at room temperature, and then analyzed by an Accuri C6 flow cytometer (BD Biosciences).

Pro-inflammatory factor levels were analysed using flow cytometry according to the instructions of LEGENDplex™ Mouse Inflammation Panel (Biolegend). Briefly, cell culture medium supernatant or RAB fractions of brain homogenate are mixed with beads and detection antibodies, and then analyzed by an Accuri C6 flow cytometer.

### Immunocytochemistry

Cells were plated on coverslips and treated as indicated. Cells were fixed with 4% paraformaldehyde and permeabilized with 0.3% Triton X-100 in PBS at room temperature. Cells were then incubated with 5% bovine serum albumin in PBS for 1 h at 37 °C and the primary antibody overnight at 4 °C, followed by three PBS washes and subsequent incubation with the fluorescent secondary antibody for 1 h at 37 °C. DAPI (1:10,000; Beyotime) was included in the final wash for nuclei counterstain. Cells were mounted with 10% glycerol and images were acquired with a laser scanning microscope (Leica SP8 Confocal System).

### Measurement of ROS

The levels of intracellular ROS were assessed by Reactive Oxygen Species Assay Kit (Beyotime), Briefly, cells were seeded in 6-well plates. After transfection of 48 h, cells were collected and incubated with DCFH-DA at 37 °C for 30 min. Samples were agitated every 3 min during incubation and immediately analyzed by flow cytometry.

### Transwell migration assay

To evaluate the chemotaxis of p-Tau induced pro-inflammatory on BV2, after 48 h of transfection of HT22, supernatants was collected and filled into the lower compartment of a transwell insert (8 µm pore size, Corning). 2.5 × 10^5^ cells were seeded in the upper compartment with serum-free medium. After 24 h, cells in the upper compartment were removed and the transwell membrane was stained with 0.5% crystal violet (Sigma-Aldrich). Migrated cells on the membrane were counted under a light microscope (Zeiss).

### Generation of knockout cell lines by CRISPR–Cas9 technology

To generate *RIPK1* knockout HT22 cells, sgRNA oligos were cloned into LentiCRISPR V2 (Addgene). 293 T cells were transfected with sgRNA vector, pPAX2 (Addgene) and pCMV–VSVg (Addgene). Supernatants containing viruses was collected 48 ~ 72 h after transfection and was supplemented with 6 µg/ml polybrene (Beyotime) to infect HT22 cells for 12–24 h. The infected cells were positively selected with 8 µg/ml puromycin to eliminate uninfected cells to generate stable cell lines and verified by western blot analysis. Similar strategies were applied to generate *RIPK3* and *MLKL* knockout cells. A nontargeting sgRNA as control. *RIPK1*, *RIPK3*, *MLKL* gRNA sequences are shown in Additional file 1: Table S1.

### Quantitative real‐time PCR

Total RNA samples were extracted using TRIzol (Invitrogen; Thermo Fisher Scientific, Inc.) and was reverse-transcribed into cDNA using according to the manufacturer’s protocol. Quantitative PCR was carried out in the CFX96 Real‐Time PCR Detection System (Bio‐Rad) using TransStart Top Green qPCR SuperMix (Transgen) according to the manufacturer's instructions. Quantitative real‐time PCR data were analyzed using the comparative Ct method, and the expression of target genes was normalized to that of GAPDH. Primer sequences are shown in Additional file 1: Table S1.

### RNA-seq

Four groups (VR1012, Tau, TauP301S, TauP301S + Nec-1) were analyzed in the transcriptome sequencing experiment. After 48 h of transfection, cells were collected. RNA degradation and contamination were monitored on 1% agarose gels. RNA purity was checked using a Nanodrop (Thermo Scientific). RNA integrity was assessed using a RNA Nano 6000 Assay Kit of the Bioanalyzer 2100 system (Agilent Technologies). After cluster generation, the library preparations were sequenced on the Illumina HiSeq platform, and 125 bp/150 bp paired-end reads were generated. The results of RNA-seq are presented in Additional file 8.

### Animals

Heterozygous transgenic mice (Tg(Prnp-MAPT*P301s)Ps19Vle/JNju) were purchased from the Model Animal Research Center of Nanjing University (Nanjing, China). The transgenes were driven by the murine prion protein (Prnp) promoter on a B6C3 background and expressed the P301S human 1N4R Tau isoform on the Chromosome 3 of mice. Genetic background and age-matched non-transgenic littermates were used as wild-type (WT) control mice. Three cohorts were employed in this monitoring experiment which included P301S male mice treated with vehicle, P301S male mice treated with Nec-1 s, and WT mice treated with vehicle in each group. Mice were treated with Nec-1 s (6.25 mg/kg) or vehicle by intraperitoneal injection twice a week as described in (Fig. 5A). Behavioral tests were implemented one week after the last injection, including body weight, hind limb clasping, nest building, novel object recognition. After behavioral tests, the mice were deeply anesthetized with pentobarbital (100 mg/kg) and perfused with phosphate-buffered saline (PBS). Half of the brain was homogenized for Western blot detection.

All animals were housed in individually ventilated cages (IVC) in Animal Experimental Platform, Core Facilities for Life Science, Jilin University. Animals were housed two or three per cage and maintained at 20–22 °C on a 12-h dark/light cycle in 40–60% humidity.

### Hindlimb clasping

Hindlimb clasping was described previously [35]. This test is scored on a scale of 0–3 which represents the best to worst phenotype. Three to four trained experimenters blinded to groups graded all mice independently every month. The average score of each mouse was calculated.

### Nest building test

The nest building test is one of the methods used to evaluate murine cognition. The nest building test started at evenfall. All mice were allocated to a single cage containing new aseptic sawdust and two pieces of 5-cm square cotton in the center of the cage, then turned off the light. Three trained experimenters blinded to groups graded all the mice independently after 24 h. The scoring rule has been described in our previous research [35]. Briefly, score 1 indicates there was no noticeable touching, score 2 indicates the cotton was partially torn up, score 3 indicates that the nest was partially complete but lower than the mouse’s head, and score 4 indicates that the nest was nearly perfect or perfect.

### Novel object recognition

Novel object recognition has been described previously [36]. On day 1, habituation phase, remove the mouse from its home cage and place it in the middle of the open, empty arena (40 cm × 40 cm × 50 cm white plastic box). Allow for free exploration of the arena for 5 min. On day 2, familiarization phase, each animal was returned to the arena containing two identical objects located in corners on the same side for 10 min. On day 3, testing phase, the animal was returned to the arena with two objects in the same positions as previously, but one object was replaced with a novel object (metal bolt and nut of similar size). Mice were allowed to explore the two objects for 10 min. Define exploration as when the mouse's noise is pointed towards the object and within 2.5 cm of the object. Thoroughly clean the apparatus between mice using 70% vol/vol ethanol. The percent exploration was determined by dividing the time exploring the novel object by the total time exploring both objects. A video analysis system (Super Maze, ZS Dichaung) was used to record the trajectory of mice.

### Mouse brain protein extraction

Right brain hemisphere of each mouse was deeply frozen in liquid nitrogen immediately after collection and transferred to − 80 °C before homogenization for Western blot detection. Brain samples were weighed and homogenized in 15 μL RAB buffer (100 mM MES, 1 mM EDTA, 2 mM dithiothreitol, 0.75 M NaCl, 0.5 mM MgSO4, pH 6.8) with protease and phosphatase inhibitor MIX (1 mM PMSF, 50 mM sodium fluoride, 1 mM sodium pyrophosphate, 1 mM sodium orthovanadate) per milligram in Bead Mill Homogenizer (Bead Ruptor 24 Elite, OMNI International) at a speed of 5.65 m/s for 45 s with a 30 s pause between two cycles. Then 1 mL brain homogenate suspension was transferred to a 1.5 mL microfuge tube (357,448, Beckman Coulter) and centrifuged at 50,000 g for 20 min at 4 °C (Optima Max-XP, Beckman Coulter). The supernatant was saved as RAB-soluble fraction (aqueous fraction). The pellet was resuspended with 1 mL RIPA buffer (150 mM NaCl, 50 mM Tris, 25 mM EDTA, 1% Triton X-100, 0.5% deoxycholic and 0.5% (w/v) SDS, pH 8.0) with protease and phosphatase inhibitor MIX and centrifuged at 50,000 g for 20 min at 4 °C to collect the supernatant as RIPA-soluble fraction (detergent soluble fraction). The pellets were further disposed by 0.5 mL urea buffer (8 M urea and 5% (w/v) SDS, pH 7.2) at 2–8 °C overnight and centrifuged to separate the supernatant as urea-soluble fraction (insoluble fraction). The three fraction samples were subpackaged in an EP tube followed by cryopreservation at − 80 °C before detection. Freeze/thaw cycles were avoided. Aliquot samples of each group were mixed together before analysis.

### Statistical analysis

Statistical analysis was performed using the commercially available GraphPad Prism program (Graphpad Software). The results are expressed as mean values ± SEM. Significant differences were assessed using unpaired two-tailed unpaired *t* tests, one-way ANOVA with Dunnett’s multiple comparisons test or two-way ANOVA with Sidak’s multiple comparisons test. Statistical significance was set at *P < 0.05, **P < 0.01, ***P < 0.001.

## Results

### Hyperphosphorylated tau activates necroptosis of neuronal cells

**Fig. 1:**
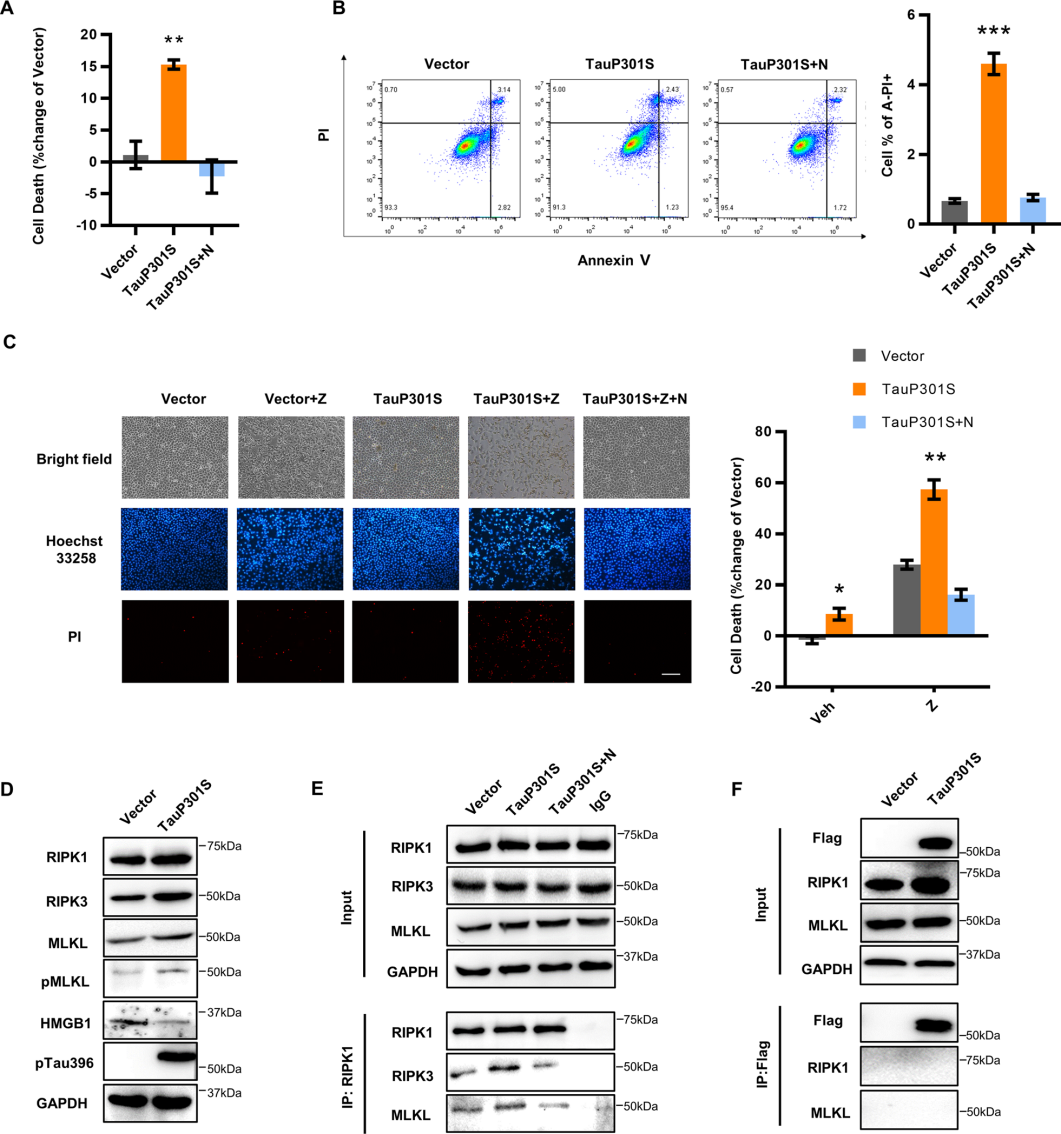
Necroptosis is activated by hyperphosphorylated tau in HT22 cells. **A, B** HT-22 cells were transfected with vector or TauP301S for 48 h. Nec-1 (30 μM) was added to the medium when the transfection medium was changed. **A** LDH was measured to assess cell death. **B** Cell death was analyzed by flow cytometry using Annexin V/PI staining. **C** Representative images of HT22 cells transfected with vector or TauP301S following treatment with DMSO or zVAD (30 μM) or zVAD (30 μM) + Nec-1 (30 μM) for 24 h, measured using Hoechst 33,258/PI staining, Scale bars, 100 μm; cell death was quantified by measuring LDH. Data are presented as the mean ± standard error of the mean (SEM) of three experiments, and a two-tailed unpaired *t* test was used to analyze the statistical significance of the data. **D** Cells were collected 48 h after transfection, and the lysates were analyzed by western blotting with the indicated antibodies. **E** HT22 cells were transfected with vector or TauP301S for 48 h. Cell lysates were immunoprecipitated with RIPK1 antibody, and the immunoprecipitated complexes were immunoblotted. **F** Immunoprecipitation and western blot analysis representative of three independent experiments. HEK 293 T cells were transfected with vector or TauP301S for 48 h. Cell lysates were immunoprecipitated with Flag beads, and the immunoprecipitated complexes were immunoblotted

To verify whether abnormally phosphorylated tau can directly lead to cell death, we transfected HEK293T cells with VR1012-TauP01S recombinant plasmid and used the VR1012-Tau construct as a control. Compared with wildtype (WT)-Tau, transfection of TauP301S induced significantly higher levels of phosphorylated tau at Ser396, 12 h after transfection (Additional file 2: Fig. S1A, B). Furthermore, marked cell death was observed concomitant with the overexpression of phosphorylated tau, suggesting that pTau can induce cell death (Additional file 2: Fig. S1C). In addition, we observed that the expression of TauP301S considerably increased the levels of RIPK1 and MLKL (Additional file 2: Fig. S1D), indicating that pTau might initiate necroptosis. We further confirmed the ability of pTau to activate necroptosis in HT22 nerve cells. The results revealed that pTau effectively induced cell death in HT22 cells; this was inhibited by Nec-1, a necroptosis inhibitor specific against RIPK1 (Fig. 1A, Additional file 2: Fig. S1E). pTau also significantly increased the number of PI^+^ annexin^−^ cells, while the number of PI^+^ annexin^+^ cells was unaltered, supporting that pTau can cause necrosis (Fig. 1B). Furthermore, the level of necroptotic cells stimulated by pTau was upregulated by the caspase inhibitor Z-VAD-FMK, which can induce TNFα-mediated necroptosis, and inhibited by the addition of Nec-1, indicating that pTau might be a direct trigger for necroptosis in neuronal cells (Fig. 1C). Like HEK293T cells, the overexpression of pTau significantly upregulated the levels of RIPK1, RIPK3, and pMLKL while downregulating intracellular high mobility group box protein 1 (HMGB1) in neuronal cell lines (Fig. 1D, Additional file 2: Fig. S1F). We also detected enhanced interactions between RIPK1, RIPK3, and MLKL after TauP301S transfection, demonstrating the formation of the necrosome induced by pTau (Fig. 1E). Nevertheless, we failed to detect a direct interaction between tau and RIPK1 or MLKL, suggesting that a specific adaptor might mediate the link between tau and necroptotic machinery (Fig. 1F). pTau-induced necroptosis activation was also detected in the neuroblastoma SH-SY5Y cell line (Additional file 2: Fig. S1G, H, I). Collectively, these data indicate that hyperphosphorylated tau can induce necroptosis in neuronal cells by activating the RIPK1–RIPK3–MLKL necroptotic machinery.

### Hyperphosphorylated tau stimulates cytokine and chemokine storms in neuronal cells

**Fig. 2:**
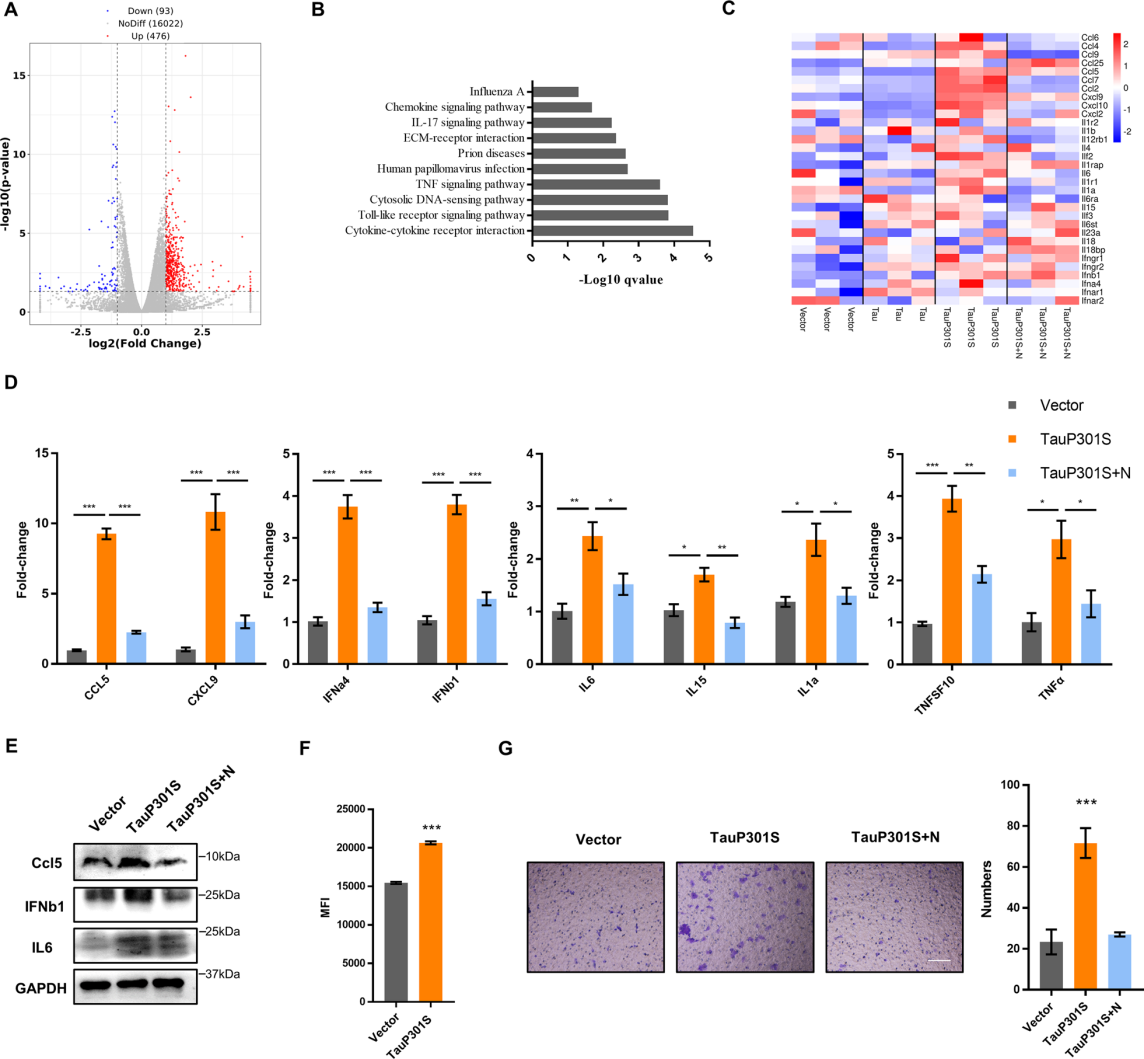
Hyperphosphorylated tau induces cytokine upregulation. **A** Differentially expressed genes (DEGs) between HT22 cells transfected with vector or TauP301S for 48 h. **B** Analysis of DEGs in the TauP301S and Tau groups according to the Kyoto Encyclopedia of Genes and Genomes (KEGG) database. **C** Heat map of cytokine genes differentially expressed in HT22 cells transfected with vector or Tau or TauP301S following treatment with DMSO or Nec-1 (30 μM); low expression is shown in blue and high expression in red. **D** mRNAs from HT22 cells transfected with vector or TauP301S following treatment with DMSO or Nec-1 (30 μM) were extracted and quantified to determine indicated cytokine levels by qPCR. **E** Cytokine levels in HT22 cells transfected with vector or TauP301S following treatment with DMSO or Nec-1 (30 μM) were analyzed by western blotting with the indicated antibodies. **F** ROS levels in HT22 cells transfected with vector or TauP301S. **G** Chemotaxis of pTau-induced cytokines on BV2 cells was analyzed by transwell assays, Scale bars, 100 μm. Data are presented as the mean ± standard error of the mean (SEM) of three experiments, and statistical analysis was performed using one-way ANOVA with Dunnett’s multiple comparisons test in **D** and two-tailed unpaired *t* test in **F, G**

To characterize transcriptional changes in necroptotic neuronal cells induced by hyperphosphorylated tau, we performed RNA-sequencing (RNA-seq) to profile the transcriptome of necroptotic HT22 cells with or without pTau overexpression. Based on the differential gene expression studies, we identified a specific transcriptional feature of neuronal death triggered by pTau, including 476 upregulated and 93 downregulated genes (Fig. 2A). KEGG (Kyoto Encyclopedia of Genes and Genomes) analysis showed that the most significantly enriched gene profiles induced by pTau were related to cytokine–cytokine receptor interaction and immune-related pathways (Fig. 2B). Coincidentally, the transcriptional levels of several pro-inflammatory cytokines, such as *IL-6*, type I interferon (IFN), and especially several chemokines, including *Ccl2*, *Ccl5*, *Cxcl9*, and *Cxcl10*, were all markedly increased, indicating that accumulated pTau stimulated the cell-autonomous transcription of pro-inflammatory cytokines and chemokines in immortalized neuronal cells (Fig. 2C). We further confirmed the expression of representative cytokines and chemokines by quantitative PCR (qPCR) and western blotting. Consistent with the RNA-seq data, the mRNA levels of *IFNa4*, *IFNb1*, *IL-6*, *IL-15*, *IL-1α*, *TNFSF10*, *TNF-α*, *Ccl5*, and *Cxcl9* were markedly increased following the overexpression of pTau (Fig. 2D). We also detected increased protein levels of IL-6, IFNβ, and Ccl5 induced by pTau (Fig. 2E, Additional file 3: Fig. S2A). Meanwhile, pTau also upregulated reactive oxygen species (ROS) in HT22 and SHSY5Y (Fig. 2F, Additional file 3: Fig. S2B). In addition, we detected enhanced secretion of pro-inflammatory cytokines, including TNF-α and IL-6, in the culture medium of HT22 cells containing accumulated pTau (Additional file 3: Fig. S2C). Chemokines are responsible for the induction of microglial chemotaxis and neuroinflammation. We noted that the induction of cytokines, such as *TNF-α* and *IL-6*, was relatively weaker than that of chemokines such as *Ccl5* and *Cxcl9* in pTau-induced necrotic neuronal cells. The results also showed that the cultured BV2 cells exhibited enhanced chemotactic ability to the cell medium of HT22 cells containing intracellular pTau (Fig. 2G). Thus, our results suggested that pTau might contribute to neuronal death by inducing cytokine and chemokine storms in neuronal cells.

### The RIPK1/RIPK3/MLKL necrosome is indispensable for cytokine stimulation driven by hyperphosphorylated tau

**Fig. 3:**
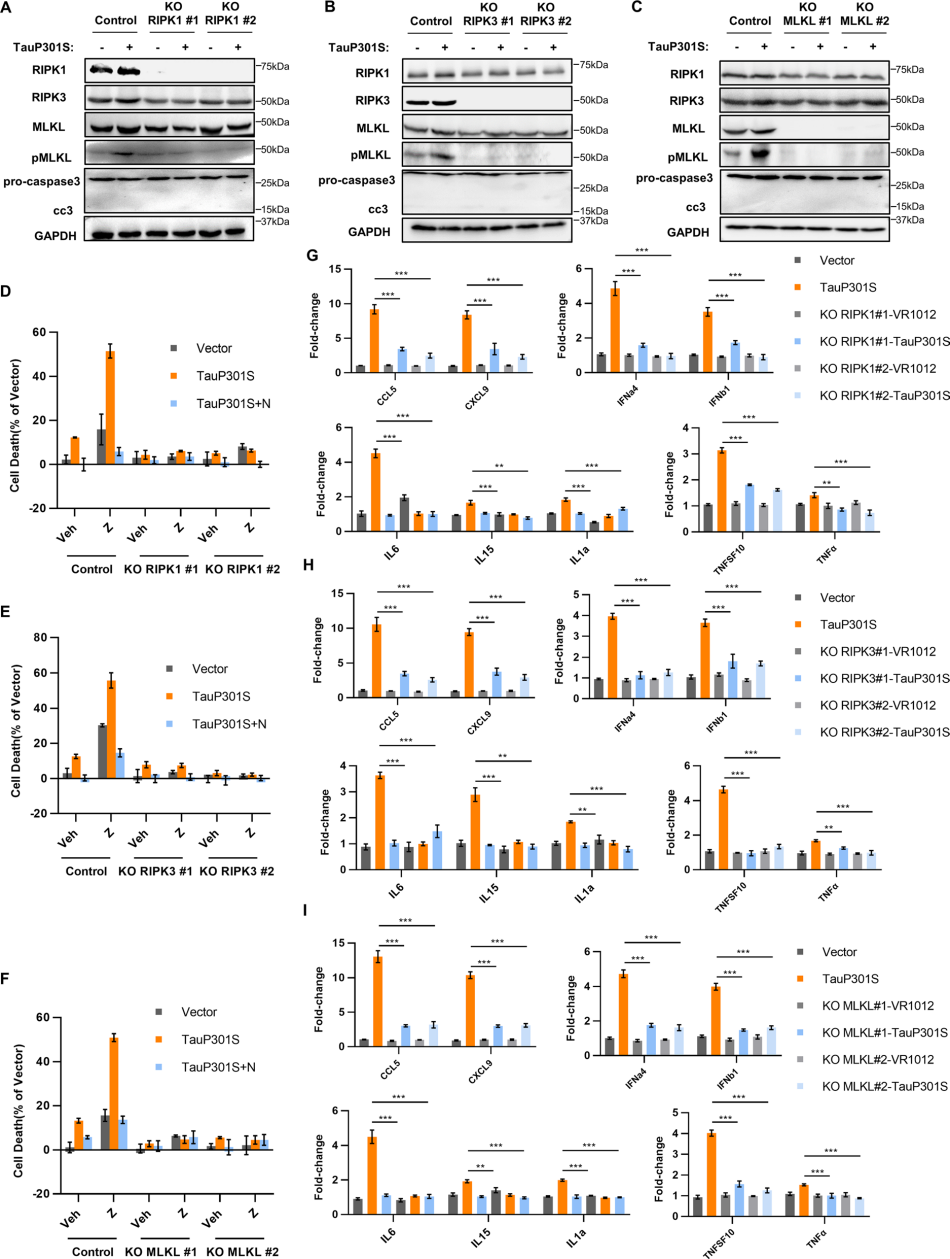
RIPK1/RIPK3/MLKL necrosome is required for hyperphosphorylated tau-stimulated cytokine induction. **A, D, G** NC HT-22 and RIPK1-KO HT22 cells were transfected with vector or TauP301S. **A** Lysates were analyzed by western blotting with the indicated antibodies. **D** LDH was assayed to determine cell death. **G** Levels of indicated cytokines were analyzed by qPCR **(B, E, H)** NC HT-22 and RIPK3-KO HT22 cells were transfected with vector or TauP301S. **B** Lysates were analyzed by western blotting with indicated antibodies. **E** LDH was assayed to examine cell death. **H** Levels of indicated cytokines were analyzed by qPCR. **C, F, I** NC HT-22 and MLKL-KO HT22 cells were transfected with vector or TauP301S. **C** Lysates were analyzed by western blotting using the indicated antibodies. **F** LDH was assayed to examine cell death. **I** Levels of indicated cytokines were analyzed using qPCR Data are presented as the mean ± standard error of the mean (SEM) of three experiments, and a two-way ANOVA with Tukey’s multiple comparisons test was used to analyze the statistical significance of the data

To further explore the involvement of necroptotic machinery in the production of cytokines driven by hyperphosphorylated tau, we applied the CRISPR–Cas9 system to stably knock out *RIPK1*, *RIPK3*, and *MLKL* in HT22 neuronal cells. On depleting *RIPK1*, *RIPK3*, and *MLKL* from cells, the level of p-MLKL was significantly decreased when compared with that in the control group (Fig. 3A–C, Additional file 4: Fig. S3). Accordingly, pTau-induced neuronal necroptosis was completely blocked by the depletion of *RIPK1*, *RIPK3*, and *MLKL* (Fig. 3D–F, Additional file 5: Fig. S4). Consistently, the production of pTau-induced pro-inflammatory cytokines and chemokines, including *IFNa4*, *IFNb1*, *IL-6*, *IL-15*, *IL-1α*, *TNFSF10*, *TNF-α*, *Ccl5*, and *Cxcl9*, was downregulated following *RIPK1*, *RIPK3*, and *MLKL* knockout. Moreover, qPCR analysis confirmed the reduced transcriptional levels of cytokines following *RIPK1*, *RIPK3*, and *MLKL* depletion in HT22 cells (Fig. 3G–I). In addition, treatment of HT22 cells with Nec-1, a specific inhibitor of RIPK1, suppressed the pTau-induced cytokine storm (Fig. 2D), indicating that the RIPK1/RIPK3/MLKL axis is critical for the overexpression of pro-inflammatory cytokines stimulated by pTau.

### The NF-κB signal pathway is essential for the induction of cytokines by hyperphosphorylated tau

**Fig. 4:**
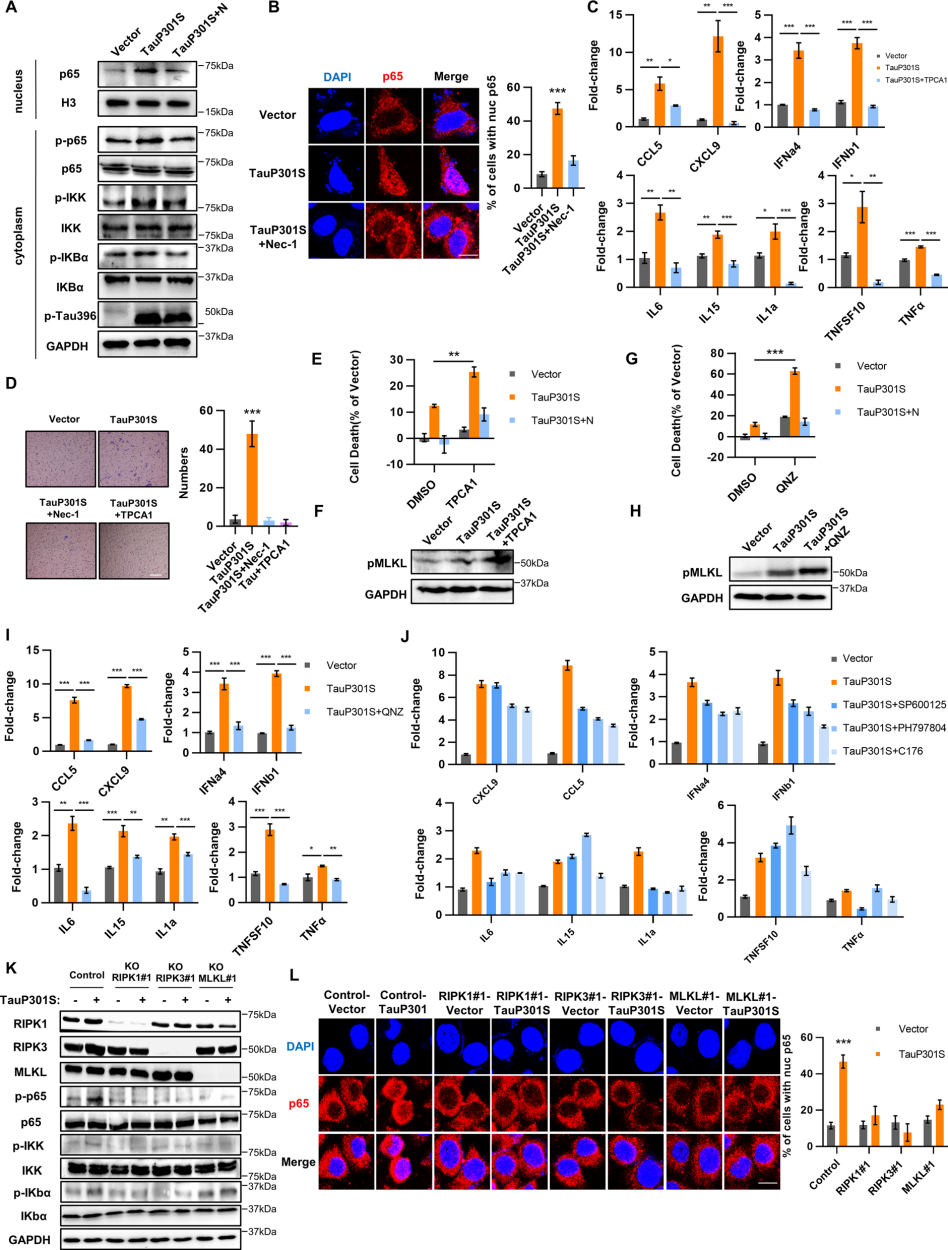
NF-κB is required for hyperphosphorylated tau-mediated cytokine induction. **A** HT22 cells were transfected with vector or TauP301S following treatment with DMSO or Nec-1 (30 μM) for 48 h, and the lysates were analyzed by western blotting using indicated antibodies. **B** Representative confocal images (left) and quantification (right) of p65 in HT22 cells transfected with vector or TauP301S following treatment with DMSO or Nec-1 (30 μM) for 48 h. Scale bars, 10 μm. **C** mRNA was extracted from HT22 cells transfected with vector or TauP301S following treatment with DMSO or TPCA1 (4 μM) and quantified to determine levels of indicated cytokines by qPCR. **D** Effect of NF-κB inhibitor on the chemotaxis of pTau-induced cytokines on BV2 cells was analyzed by transwell assays, Scale bars, 100 μm. **E, F, I** HT22 cells were transfected with vector or TauP301S following treatment with DMSO or TPCA1 (4 μM) or TPCA1 (4 μM) + Nec-1 (30 μM) for 48 h; **E** cell death was measured measuring LDH levels; **F** levels of the indicated cytokines were analyzed using qPCR; **I** lysates were analyzed by western blotting using indicated antibodies. **G, J** HT22 cells were transfected with vector or TauP301S following treatment with DMSO or QNZ (5 μM) or QNZ (5 μM) + Nec-1 (30 μM) for 48 h. **G** Cell death was evaluated by measuring LDH levels; **J** lysates were analyzed by western blotting using indicated antibodies. **H** mRNA was extracted from HT22 cells transfected with vector or TauP301S following treatment with DMSO or SP600125 (5 μM), PH797804 (5 μM), or C176 (2 μM), followed by quantification to determine levels of the indicated cytokines by qPCR. Data are presented as the mean ± standard error of the mean (SEM) of three experiments, statistical analysis was performed using two-tailed unpaired *t* test in **E, G** and one-way ANOVA with Dunnett’s multiple comparisons test in **C, F**. **K** NC HT22, RIPK1-KO HT22, RIPK3-KO HT22, and MLKL-KO HT22 cells were transfected with vector or TauP301S, and lysates were analyzed by western blotting using indicated antibodies. **L** Representative confocal images (left) and quantification (right) of p65 in NC HT22, RIPK1-KO HT22, RIPK3-KO HT22, and MLKL-KO HT22 cells transfected with vector or TauP301S. Scale bars, 10 μm. Data are presented as the mean ± standard error of the mean (SEM) of three experiments, and statistical analysis was performed using two-tailed unpaired *t* test in **B**, **L**

Previous studies have shown that the NF-κB pathway is required for cytokine expression during TNFα-induced necroptosis. We next examined whether the NF-κB pathway was also involved in the pTau-induced cytokine storm. Upon transfection of TauP301S in HT22 cells, the level of phosphorylated IκB significantly increased, accompanied by the upregulated p65 phosphorylation (Fig. 4A, Additional file 6: Fig. S5A). Meanwhile, we detected a significant nuclear accumulation of p65 (Fig. 4A, B), a critical event for NF-κB, after Tau phosphorylation, confirming the pTau-induced activation of the NF-κB signaling pathway. We then applied different inhibitors of the NF-κB pathway to determine the impact of NF-κB on pTau-induced cytokine expression. Herein, we observed that blocking NF-κB activation by adding the IKK inhibitor TPCA-1 abrogated cytokine induction comprehensively in HT22 cells, suggesting that NF-κB activation is essential for pTau-induced cytokine production (Fig. 4C). In addition, TPCA1 inhibited the enhanced chemotactic ability to the cell medium of HT22 cells containing intracellular pTau (Fig. 4D). However, we observed that TPCA-1 treatment could not abolish hyperphosphorylated tau-induced necroptosis. Instead, the inhibition of IKK promoted neuronal necroptosis in HT22 cells (Fig. 4E, F). These data are consistent with the previous finding that IKK can phosphorylate and inhibit RIPK1, and IKK suppression upregulated the level of phosphorylated RIPK1. Accordingly, we further applied another inhibitor of NF-κB pathway to evaluate the impact of suppressing the NF-κB signal on neuronal necroptosis. The results revealed that the inhibition of NF-κB suppressed cytokine induction and promoted neuronal necroptosis in HT22 cells (Fig. 4G, H, I). We also evaluated the potential roles of STING and MAPKs, such as p38 and JNK, during cytokine induction. Compared with the effect of the NF-κB inhibitor TPCA-1, the P38 inhibitor PH797806, JNK inhibitor SP600125, and STING inhibitor C176 weakly influenced pTau-mediated cytokine induction (Fig. 4J), suggesting that activation of the canonical NF-κB pathway might be the predominant mediator of cytokine induction during necroptosis of neuronal cells.

### The RIPK1–RIPK3–MLKL axis regulates NF-κB activation during hyperphosphorylated tau-induced necroptosis

We next determined the role of the RIPK1/RIPK3/MLKL complex in activating the NF-κB pathway during pTau-mediated cytokine induction in neuronal cells. In HT22 cells, depletion of any RIPK1/RIPK3/MLKL necrosome component suppressed the activation of the NF-κB pathway induced by pTau. Furthermore, the abolishment of RIPK1, RIPK3, and MLKL not only decreased the level of phosphorylated IκB but also inhibited p65 phosphorylation (Fig. 4K, Additional file 6: Fig. S5B). In addition, the nuclear translocation of p65 failed to occur after depletion of any necrosome protein, indicating the critical role of the RIPK1/RIPK3/MLKL necrosome for pTau-mediated activation of the NF-κB pathway (Fig. 4L). Similarly, treatment with Nec-1, a RIPK1 kinase inhibitor, significantly downregulated pTau-stimulated phosphorylated p65 levels (Fig. 4A). Based on these findings, we concluded that the RIPK1–RIPK3–MLKL axis is specifically required for activating NF-κB during pTau-induced cytokine storm. pTau could trigger neuronal death through two synergetic signaling pathways, including the RIPK1/RIPK3/MLKL necrosome and the RIPK1–RIPK3–MLKL–NF-κB axis.

### Necroptosis and cytokine expression are both activated in TauP301S transgenic mice, and blocking necroptosis improves behavioral defects and neuroinflammation efficiently in an AD mice model

**Fig. 5:**
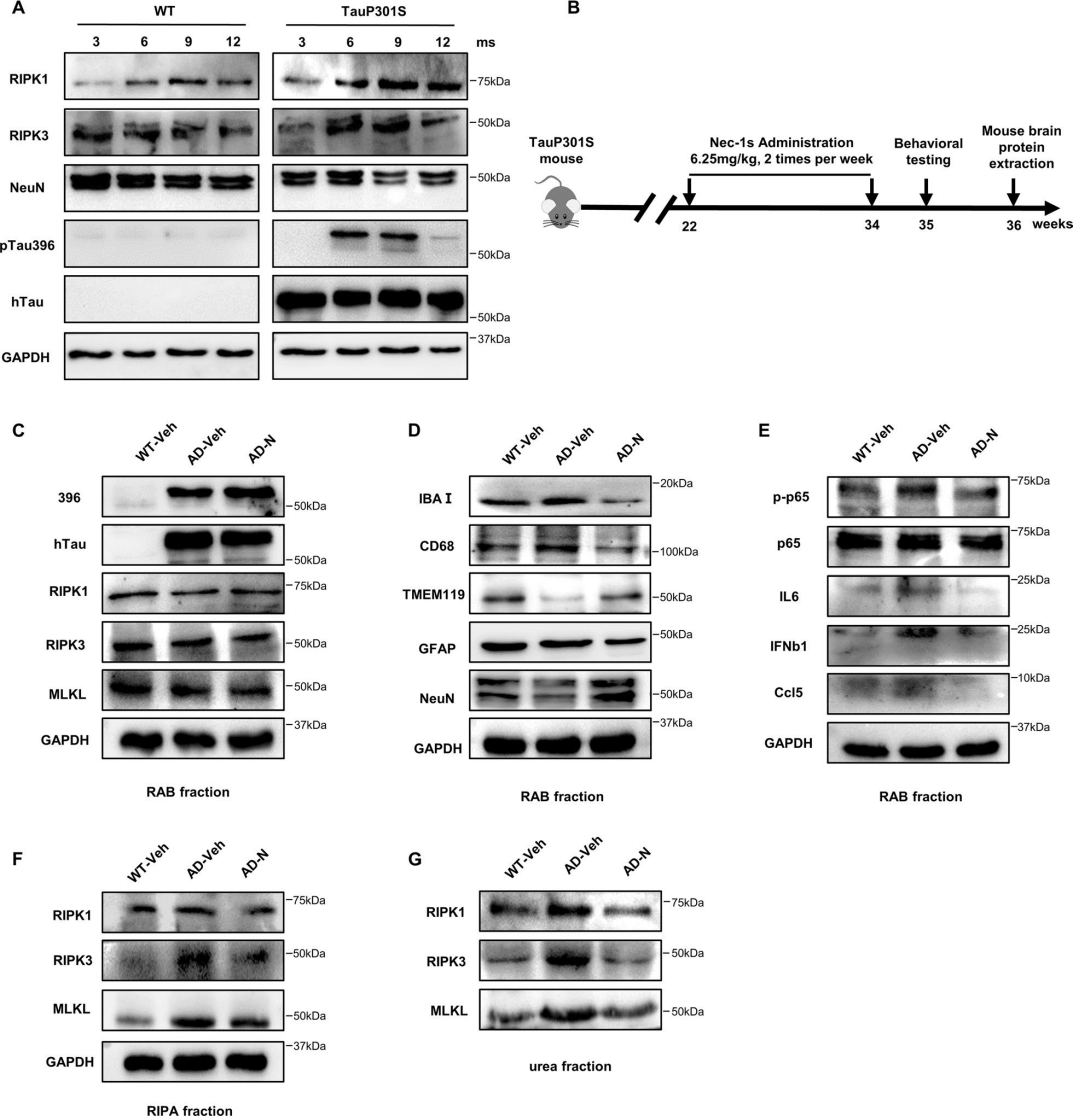
Nec-1 s improves neuroinflammation and neuronal cell death in an AD mouse model. **A** RAB fraction of mouse brain homogenates at 3, 6, 9, 12 months of age was analyzed by western blotting using indicated antibodies (*n* = 7). **B** 5.5-month-old male TauP301S mice were intraperitoneal injection Nec-1 s (6.25 mg/kg) or vehicle (45% PEG400, 2.8% DMSO in PBS) for 12 weeks (2 times per week, *n* = 8–9). Age-matched male wild-type (WT) mice were injected with vehicle (45% PEG400, 2.8% DMSO in PBS) for 12 weeks, (*n* = 8–9). **C–E** RAB fraction of mouse brain homogenates was analyzed by western blotting with the indicated antibodies (*n* = 8–9). **F** RIPA fraction of mouse brain homogenates was analyzed by western blotting using indicated antibodies (*n* = 8–9). **G** Urea fraction of mouse brain homogenates was analyzed by western blotting using indicated antibodies (*n* = 8–9)

To investigate the role of necroptosis in tauopathies, we examined neuronal death in the brain of a TauP301S AD mouse model at different ages. Compared with the WT mice, pTau was significantly accumulated in the brain homogenate of TauP301S transgenic mice at 6 months of age and persistently expressed at a high level until 9 months. In parallel with exacerbated tauopathies, the number of neuronal cells substantially decreased in AD mice from 9 months of age. Furthermore, RIPK1 began accumulating in the brain of TauP301S mice at 3 months, and its level was higher than that in WT mice. RIPK1 was abundant at 6 months of age and increased significantly by 9 months of age. Similarly, RIPK3 began accumulating at 6 months and reached an expression peak at 9 months of age (Fig. 5A, Additional file 7: Fig. S6A). Thus, the results from TauP301S mice strongly confirmed that pTau activates neuronal necroptosis and that the onset of necroptosis proceeds in an age-dependent manner in AD mice with tau pathology.

Next, we treated the TauP301S mice with Nec-1 s from 5.5 months to 8.5 months of age and then compared their neuropathologic and behavioral phenotypes with those of control mice (Fig. 5B). As expected, Nec-1 s treatment reduced the RIPK1, RIPK3 and MLKL level in the insoluble fraction of brain homogenates derived from transgenic mice but not in the soluble fraction (Fig. 5C, F, G, Additional file 7: Fig. S6F). Our previous studies have shown that in TauP301S mice, pTau induces gradual microglial accumulation and conversion to an active state. Furthermore, treatment with Nec-1 s downregulated the IBa1 and CD68 level and restored the NeuN and TMEM119 level in the brain of TauP301S mice, indicating that that Nec-1 s inhibited pTau-induced neuronal death and microglia hyperactivation (Fig. 5D, Additional file 7: Fig. S6B). In addition, we evaluated expression levels of cytokines and chemokines in the brains of TauP301S mice after Nec-1 s treatment. As shown, at 9 months of age, cytokine levels, including IL-6, IFNβ and Ccl5, were significantly higher in non-treated AD mice than those in WT mice, demonstrating that neurotoxic Tau could also induce cytokine storm in AD mice (Fig. 5E, Additional file 7: Fig. S6C–E). Strikingly, Nec-1 s treatment markedly downregulated the expression of cytokines, supporting our in vitro findings that the RIPK1/RIPK3/MLKL necrosome is responsible for pTau-mediated cytokine induction.

**Fig. 6:**
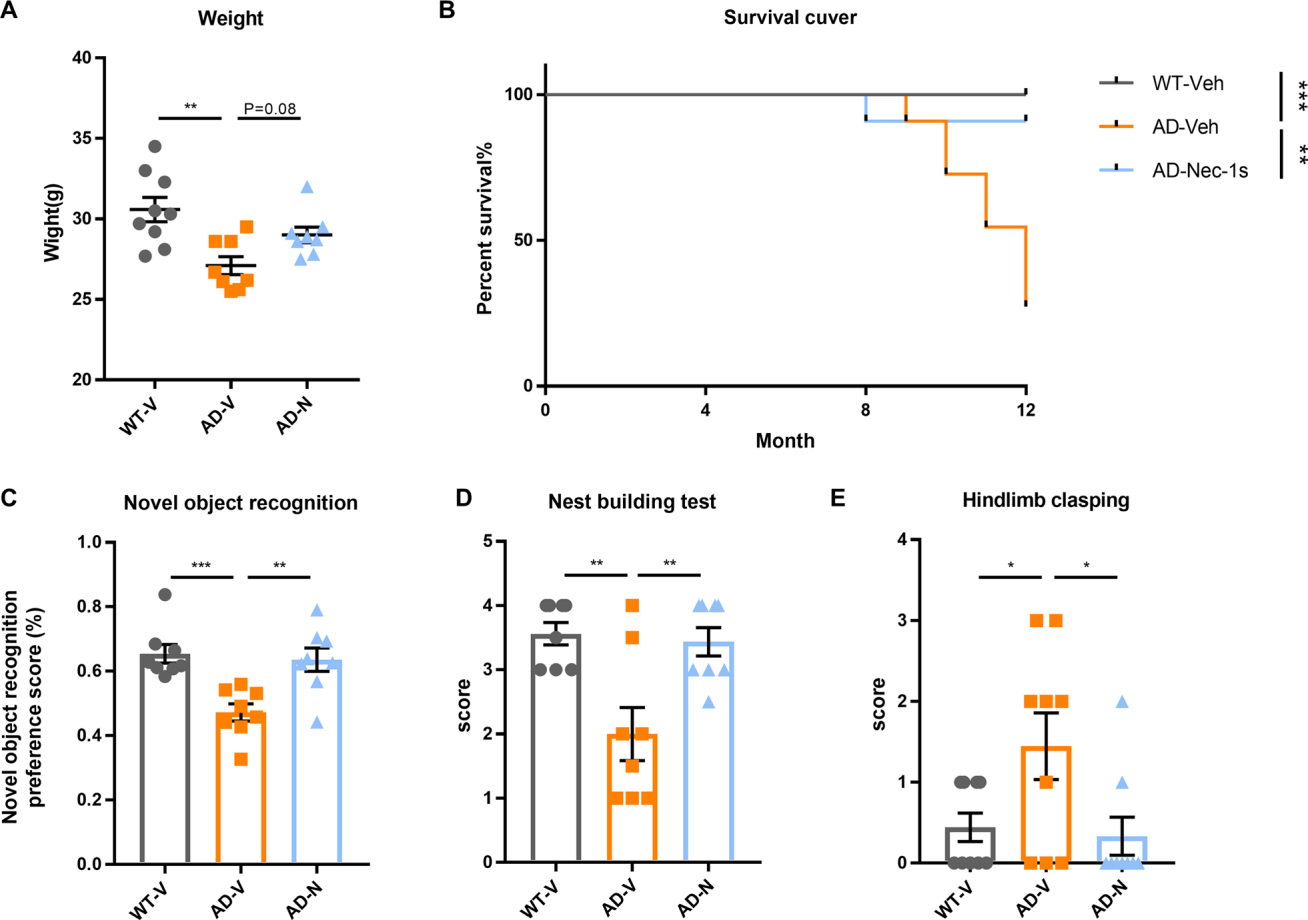
Nec-1 s improves behavioral defects in an AD mouse model. **A** Body weight of mice 1 week after the last injection in the three groups (*n* = 8–9). **B** Kaplan–Meier survival analysis of three mouse groups injected with Nec-1 (6.25 mg/kg) or vehicle (*n* = 11 per group). **C** Percentage exploration in three mouse groups in the novel object recognition paradigm (*n* = 8–9 per group). **D** Nest-building test was scored after 24 h, immediately after equal amounts of cotton were placed in each cage and lights were turned off (*n* = 8–9 per group). **E** Hindlimb clasping scores of three mouse groups on performing the hindlimb clasping test. (*n* = 8–9 per group). Data are presented as mean ± standard error of the mean (SEM), and one-way ANOVA with Dunnett’s multiple comparisons test was used to analyze the statistical significance of the data

We further determined whether the inhibition of necroptosis could alleviate behavioral defects in TauP301S mice. Compared with WT mice, the body weight of transgenic mice markedly declined with age. Notably, Nec-1 s treatment restored the decrease in body weight in transgenic mice (Fig. 6A). In addition, treatment with Nec-1 s significantly prolonged the survival rate of TauP301S mice (Fig. 6B). Furthermore, TauP301S mice exhibited remarkably improved learning and cognitive ability in the novel object recognition paradigm after Nec-1 s treatment (Fig. 5C). The nest-building test is sensitive to hippocampal damage and can be employed as a mouse model of mental diseases. We observed that the nesting ability of transgenic mice was notably impaired when compared with that of WT mice, and treatment with Nec-1 s significantly improved the nesting ability of Tau model mice (Fig. 6D). In addition, hind limb clasping was used to measure the muscle atrophy phenotype of treated TauP301S and control mice (Fig. 6E). The results revealed that hind limbs of transgenic mice were easier to clamp than those of WT mice, and the Nec-1 s-treated group exhibited similar performance to the WT group. Collectively, our data demonstrated that neuronal necroptosis and cytokine expression were both stimulated in an AD mouse model exhibiting typical tau pathology, and suppression of necroptosis markedly ameliorated the behavioral decline in this mouse model.

## Discussion

**Fig. 7:**
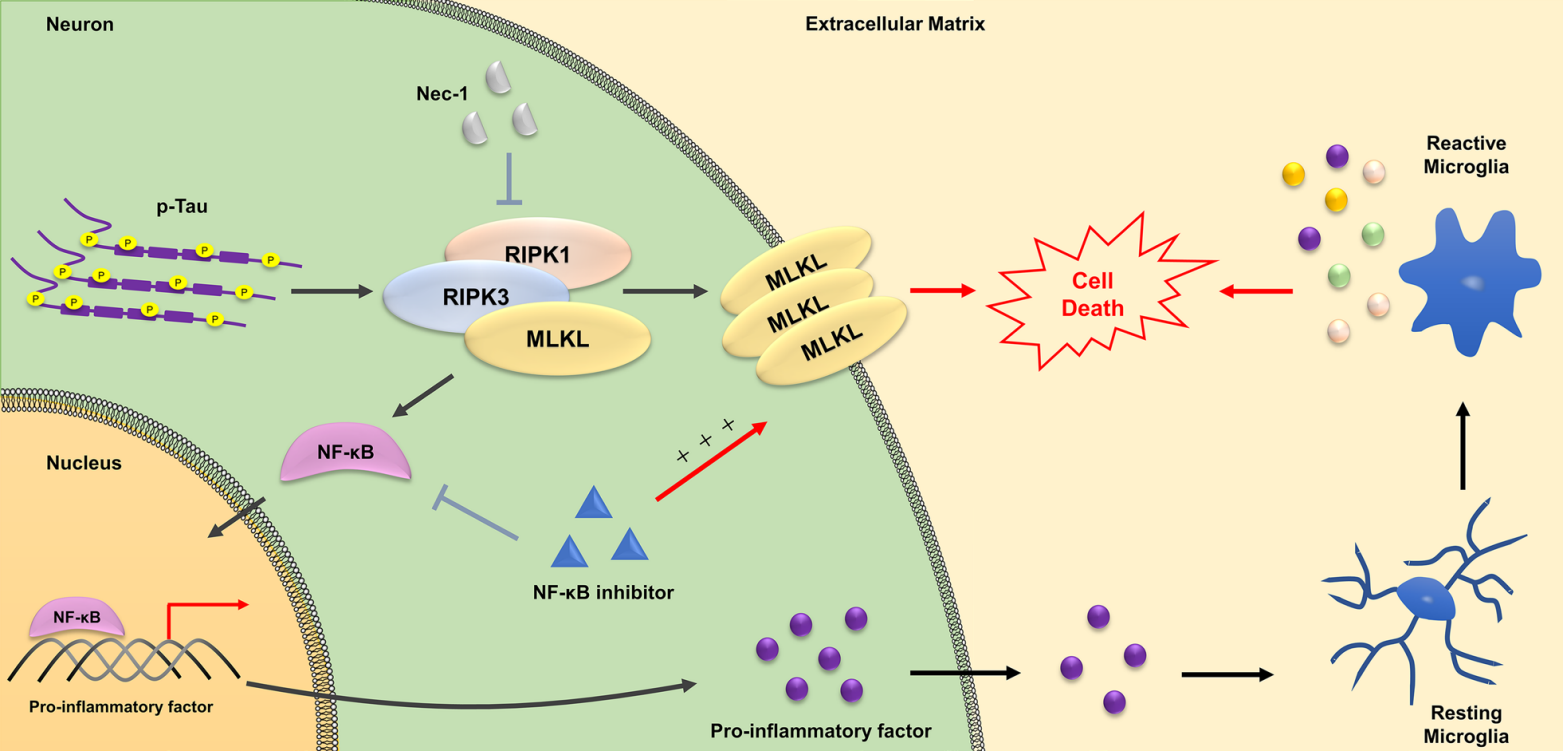
Hyperphosphorylated tau contributes to v by inducing necroptosis and inflammation mediated by activating the RIPK1/RIPK3/MLKL and NF-κB pathways. Hyperphosphorylated tau promotes necroptosis by activating RIPK1/RIPK3/MLKL axis. Meanwhile, necroptosis is accompanied by the up regulation of pro-inflammatory factors transcription mediated by NF-κB pathway. Furthermore, microglia is activated by pro-inflammatory factors and jointly lead to neuronal cells death. Necroptosis and pro-inflammatory factors induction could be blocked by inhibition of RIPK1. However, NF-κB inhibitors can down regulate the transcription of pro-inflammatory factors, but promote necroptosis

AD is a progressive neurodegenerative disease characterized by progressive neuronal loss in several brain regions. It is associated with cognitive and behavioral deficits owing to the atrophy of affected areas. However, there is a lack of knowledge regarding the primary contributors and detailed mechanisms underlying neuronal death in AD. Currently, it is evident that the “amyloid cascade hypothesis” fails to comprehensively explain the neuronal loss in AD, as verified by human autopsy and imaging studies. In addition to amyloid plaques, intraneuronal NFTs constituted by pTau protein are key pathological features of AD. It is well-established that aggregated pTau results in microtubule destabilization, defective axonal transport, and neuronal damage; however, the precise mechanism through which this devastating pathology ultimately affects neuronal death remains unclear. Studies in human AD brains have shown that necroptosis activation is triggered by the RIPK1/RIPK3/MLKL complex and contributes to chronic neuronal death in AD. Although their results suggested that tau accumulation might be a key trigger for necroptosis activation in AD, the precise mechanism regulating RIPK1 activation in AD remains unclear. In the present study, we reported the first evidence that pTau is the key initiator of necroptosis in cultured neuronal cells, as well as in a mouse model of AD that develops tau pathology. In addition, pTau can stimulate cytokine storms in neuronal cells via the NF-κB signaling pathway, and the RIPK1–RIPK3–MLKL axis is specifically required for both these processes. Furthermore, inhibiting necroptosis remarkably ameliorated the behavioral deficits in an AD mouse model harboring NFT. Thus, our study demonstrates, for the first time, that pTau contributes to neuronal death primarily by activating necroptosis and inducing cytokine expression (Fig. 7). In addition, these results strongly support that targeting pTau might be a more effective therapeutic strategy for AD.

The proximal event of neuronal loss in AD is an actively debated issue that has mainly focused on two hallmark lesions of AD, including the extracellular deposited senile plaques formed by aggregated Aβ and intraneuronal accumulated NFTs composed of pTau protein. The pivotal role of Aβ protein during the progression of AD is supported by the evidence that genetic mutations of APP cause familial AD, as well as by the finding that Aβ peptides exhibit toxicity toward neurons in vitro and in vivo [2, 29]. One potential mechanism of Aβ neurotoxicity is that aggregated Aβ can induce apoptosis by interacting with neuronal receptors and trigger signal transduction cascades that lead to caspase activation and oxidative stress induction [30, 31]. Despite the ability of Aβ aggregates to induce apoptosis, the contribution of apoptosis to neuronal loss in AD remains unclear despite several reports [32]. First, imaging studies and human autopsy examinations indicated that deposited Aβ plaques could also be detected in cognitively normal elderly individuals. Second, although several immunotherapies targeting Aβ cleared Aβ plaques efficiently in both animal and human models, they still failed to modify the progression of AD. Moreover, AD is a chronic, slowly progressing neurodegenerative disease rather than an acute disorder. Moreover, a large number of damaged neurons fail to demonstrate morphological features of apoptosis, despite constant exposure to apoptotic stimuli, suggesting that Aβ-induced apoptosis might not account for neuronal death in AD.

NFTs are another key pathological feature of AD, primarily composed of paired helical filaments consisting of pTau. Typically, Tau tangles begin to occur in the entorhinal cortex and hippocampus and then spread to the temporal-associated cortex [33]. The occurrence of neuronal death generally parallels NFT formation, and the clinical features and severity of AD better correlate with NFT pathology than Aβ deposition [18]. Although tau pathology results in axonal transport defects, oxidative stress, and ultimately neuronal death, the mechanism through which pTau promotes neuronal death has not been thoroughly examined. Caccamo et al. were the first to report that the RIPK1/RIPK3/MLKL necrosome is activated and contributes to neuronal loss in human AD brains and an AD mouse model that develops neuronal death. Importantly, their results strongly suggested a positive correlation between neuronal necroptosis and pTau, indicating that pathological tau is more proximal than Aβ aggregates to neuronal death in AD. Nevertheless, there is currently no report verifying the function of pTau in triggering neuronal necroptosis and the underlying mechanisms. Herein, we demonstrated that pTau directly contributes to necroptosis in cultured neuronal cell lines, as well as in a mouse model of AD that develops tau pathology. Overexpression of pTau significantly upregulated the levels of phosphorylated RIPK1, RIPK3, and MLKL and increased the formation of the RIPK1/RIPK3/ MLKL necrosome. Furthermore, knockout of RIPK1, RIPK3, and MLKL abolished pTau-induced necroptosis, strongly demonstrating that pTau is a direct trigger for necroptosis activation in AD. However, we failed to detect any direct interaction between Tau and necrosome proteins, suggesting that a specific adaptor or event might act as a mediator between pathological tau and the necroptotic machinery. Future research is needed to explore the underlying mechanism via which pTau induces necroptosis.

Neuroinflammation plays a crucial role in neurodegenerative diseases, whereas the potential mechanism of neuroinflammation induction and the influence of inflammatory factors on disease progression remain unclear. Several studies have suggested that microglial activation and the subsequent release of pro-inflammatory cytokines play important roles in neuronal degeneration [20]. Conversely, Zhu et al. have demonstrated that necroptosis could promote the cell-autonomous production of pro-inflammatory cytokines [27]. In addition, Ito et al. have reported that optineurin (OPTN) deficiency leads to axonal degeneration in ALS by inducing inflammation and necroptosis, which is mediated by RIPK1/RIPK3/MLKL necroptotic machinery [28]. Collectively, these findings indicate that elevated cytokine expression might be a common feature of necroptosis in different cells and can be activated by diverse necroptotic triggers. Reportedly, necroptosis is activated in postmortem human AD brains and positively correlates with the Braak stage. However, in the context of AD, it needs to be determined whether neuronal necroptosis could promote cytokine production. Moreover, are pro-inflammatory cytokines released by neurons themselves or classical microglia? Finally, can any specific stimuli induce necroptosis-mediated cytokine expression during neuronal death? All these questions remain to be addressed. In the present study, we provided compelling evidence that pTau could positively regulate neuronal necroptosis in both cultured neuronal cell lines and an AD mouse model. Meanwhile, pTau promoted cell-autonomous activation of pro-inflammatory cytokine expression, and the released chemokine could further activate microglia, leading to persistent neuroinflammation. In addition, our results demonstrated that the RIPK1/RIPK3/MLKL necrosome is responsible for pTau-stimulated cytokine storms. Consistent with the findings of Yuan et al., our work also confirmed that NF-κB is required for the pTau-induced cytokine transcription in neuronal cells. In contrast, we revealed that during pTau-induced chronic neuronal death, IκB phosphorylation and the nuclear translocation of p65 are both modulated by the RIPK1–RIPK3–MLKL axis, indicating that necroptotic machinery protein likely acts upstream of IKK to directly promote IKK phosphorylation and IκBα degradation, and finally initiating the activation of NF-κB during neuronal necroptosis in AD. Further studies are needed to clarify the detailed mechanism through which the necroptotic machinery regulates the NF-κB signaling pathway in neurodegenerative disorders.

## Conclusions

Overall, we provide the first direct evidence confirming that pTau is the key trigger for neuronal death by inducing necroptosis and inflammation in AD. These findings reveal new insights into the mechanisms underlying necroptosis activation in AD, as well as highlight the critical role of neurotoxic tau in the pathogenesis of this disorder. Accordingly, therapeutics aimed to clear pathological tau might be a more potent strategy to halt or impede the progression of AD.

## Supplementary Information

**Additional file 1: Table S1.** sgRNAs and qPCR primer sequences.

**Additional file 2: Figure S1.** Necroptosis was stimulated by hyperphosphorylated tau. (A) HEK 293 T cells were transfected with TauP301S for 0, 12, 24, 48 h and the lysates were analyzed by western blotting with AT8. (B) HEK 293 T cells were transfected with 0.5, 1, 2 or 4 μg TauP301S and the lysates were analyzed by western blotting using indicated antibodies. (C) Representative images of HEK 293 T cells transfected with vector or TauP301S in bright field, Scale bars, 50 μm. (D) HEK 293 T cells were transfected with 0.5, 1, 2 or 4 μg TauP301S and the lysates were analyzed by western blotting using indicated antibodies, quantification of the immunoreactivity of the blots, normalized against GAPDH (E) Representative images of HT22 cells transfected with vector or TauP301S, followed by treatment with DMSO or Nec-1 (30 μM) for 24 h and examined by Hoechst 33258/PI staining, Scale bars, 100 μm. (F) Quantification of the immunoreactivity of the blots in Fig. 1D, normalized against GAPDH. (G) SH-SY5Y cells were transfected with vector or TauP301S, followed by treatment with DMSO or Nec-1 (30 μM) for 48 h; cell death was analyzed by flow cytometry using Annexin V/PI staining. (H) SH-SY5Y cells were transfected with vector or TauP301S, and the lysates were analyzed by western blotting using indicated antibodies. (I) Representative images of HT22 cells transfected with vector or TauP301S, followed by treatment with DMSO or zVAD (30 μM) or zVAD (30 μM) + Nec-1 (30 μM) for 24 h and examined by Hoechst 33258/PI staining, Scale bars, 10 μm; cell death was quantified by measuring LDH levels. Data are presented as the mean ± standard error of the mean (SEM) of three experiments, and statistical analysis was performed using two-way ANOVA with Tukey’s multiple comparisons test in D and two-tailed unpaired *t* test in F, G, I.

**Additional file 3: Figure S2.** Hyperphosphorylated tau upregulated reactive oxygen species (ROS) and cytokine level in neuronal cells. (A) Quantification of the immunoreactivity of the blots in Fig. 2E, normalized against GAPDH. (B) ROS levels in SH-SY5Y transfected with vector or TauP301S were quantified by flow cytometry. (C) Secretion of TNF-α and IL-6 was quantified using flow cytometry. Data are presented as mean ± standard error of the mean (SEM) of three experiments, and statistical analysis was performed using one-way ANOVA with Dunnett’s multiple comparisons test in A and two-tailed unpaired *t* test in B, C.

**Additional file 4: Figure S3.** Hyperphosphorylated tau induces necroptosis in HT22 requiring RIPK1, RIPK3 and MLKL. (A) Quantification of the immunoreactivity of the blots in Fig. 3A, normalized against GAPDH. (B) Quantification of the immunoreactivity of the blots in Fig. 3B, normalized against GAPDH. (C) Quantification of the immunoreactivity of the blots in Fig. 3C, normalized against GAPDH. Data are presented as mean ± standard error of the mean (SEM) of three experiments, and a two-way ANOVA with Sidak's multiple comparisons test was used to analyze the statistical significance of the data.

**Additional file 5: Figure S4.** Knockdown of RIPK1, RIPK3 and MLKL inhibits hyperphosphorylated Tau-induced necroptosis. Representative images of NC, RIPK1-KO, RIPK3-KO and MLKL-KO cells transfected with vector or TauP301S following treatment with DMSO or zVAD (30 μM) or Nec-1 (30 μM) or zVAD (30 μM) + Nec-1 (30 μM) for 24 h, measured using Hoechst 33258/PI staining, Scale bars, 100 μm.

**Additional file 6: Figure S5.** NF-κB signalling pathway is regulated by the RIPK1–RIPK3–MLKL axis. (A) Quantification of the immunoreactivity of the blots in Fig. 4A. (B) Quantification of the immunoreactivity of the blots in Fig. 4K. Data are presented as mean ± standard error of the mean (SEM) of three experiments, and statistical analysis was performed using one-way ANOVA with Dunnett’s multiple comparisons test in A and two-way ANOVA with Sidak's multiple comparisons test in B.

**Additional file 7: Figure S6.** Nec-1 s treatment reduces neuroinflammation in TauP301S mice. (A) Quantification of the immunoreactivity of the blots in Fig. 5A, normalized against GAPDH (*n* = 7). (B) Quantification of the immunoreactivity of the blots in Fig. 5D, normalized against GAPDH (*n* = 8–9). (C) mRNAs from mice brain were extracted and quantified to determine indicated cytokine levels by qPCR. (D) Analysis of pro-inflammatory factors and chemokines in RAB fractions by flow cytometry. (E) Quantification of the immunoreactivity of the blots in Fig. 5E, normalized against GAPDH (*n* = 8–9). (F) Quantification of the immunoreactivity of the blots in Fig. 5F, normalized against GAPDH (*n* = 8–9).Data are presented as mean ± standard error of the mean (SEM) of three experiments, and statistical analysis was performed using two-way ANOVA with Sidak's multiple comparisons test in A and one-way ANOVA with Dunnett’s multiple comparisons test in B, C, D, E, F.

**Additional file 8.** Expression of RNA-seq.

**Additional file 9.** Raw images of Western blot.

## Data Availability

All data are available from the corresponding author upon reasonable request.

## Abbreviations

AD: Alzheimer’s disease
Aβ: Amyloid-β
APP: Amyloid beta precursor protein
Ccl: C–C Motif Chemokine Ligand
Cxcl: C–X–C motif chemokine ligand
HMGB1: High mobility group box protein 1
IL: Interleukin
IKK: Inhibitor of nuclear factor kappa B Kinase
IκB: Inhibitor of nuclear factor kappa-B
Interferon: IFN
KEGG: Kyoto encyclopedia of genes and genomes
MAPT: Microtubule-associated protein
MLKL: Mixed lineage kinase domain-like protein
MAPK: Mitogen-activated protein kinase
NFTs: Neurofibrillary tangles
NF-κB: Nuclear factor kappa B
pTau: Hyperphosphorylated tau
RIPK: Receptor-interacting protein
ROS: Reactive oxygen species
STING: Stimulator of interferon genes
TNF: Tumor necrosis factor
WT: Wild-type
