# Supplementary material for: Hyperphosphorylated tau mediates neuronal death by inducing necroptosis and inflammation in Alzheimer’s disease
Source: J Neuroinflammation. 2022 Aug 15;19:205. doi: 10.1186/s12974-022-02567-y (PMC9377071; doi:10.1186/s12974-022-02567-y)
Supplement: Supplementary file 1 — Additional file 1: Table S1. sgRNAs and qPCR primer sequences. [file 12974_2022_2567_MOESM1_ESM.doc]

Additional Information for:

**Hyperphosphorylated tau mediates neuronal death by inducing necroptosis and inflammation in Alzheimer’s disease**

Yue Dong^1^, Hanqiao Yu^1^, Yayuan Zheng^1^, Mingrui Dai^1^, Xuejian Feng^1^, Yao Sun^1^, Yu He^1^, Bin Yu^1, 2^, Haihong Zhang^1, 2^, Jiaxin Wu^1, 2^, Xianghui Yu^1, 2^, Hui Wu^1, 2*^, Wei Kong^1, 2*^

**Figure legends**

**Additional Fig. S1 Necroptosis was stimulated by hyperphosphorylated tau**

**(A)** HEK 293T cells were transfected with TauP301S for 0, 12, 24, 48 h and the lysates were analyzed by western blotting with AT8. **(B)** HEK 293T cells were transfected with 0.5, 1, 2 or 4 μg TauP301S and the lysates were analyzed by western blotting using indicated antibodies. **(C)** Representative images of HEK 293T cells transfected with vector or TauP301S in bright field, Scale bars, 50 μm. **(D)** HEK 293T cells were transfected with 0.5, 1, 2 or 4 μg TauP301S and the lysates were analyzed by western blotting using indicated antibodies, quantification of the immunoreactivity of the blots, normalized against GAPDH **(E)** Representative images of HT22 cells transfected with vector or TauP301S, followed by treatment with DMSO or Nec-1 (30 μM) for 24 h and examined by Hoechst 33258/PI staining, Scale bars, 100 μm. **(F)** Quantification of the immunoreactivity of the blots in figure 1D, normalized against GAPDH. **(G)** SH-SY5Y cells were transfected with vector or TauP301S, followed by treatment with DMSO or Nec-1 (30 μM) for 48 h; cell death was analyzed by flow cytometry using Annexin V/PI staining. **(H)** SH-SY5Y cells were transfected with vector or TauP301S, and the lysates were analyzed by western blotting using indicated antibodies. **(I)** Representative images of HT22 cells transfected with vector or TauP301S, followed by treatment with DMSO or zVAD (30 μM) or zVAD (30 μM)+Nec-1 (30 μM) for 24 h and examined by Hoechst 33258/PI staining, Scale bars, 10 μm; cell death was quantified by measuring LDH levels. Data are presented as the mean ± standard error of the mean (SEM) of three experiments, and statistical analysis was performed using two-way ANOVA with Tukey’s multiple comparisons test in **D** and two-tailed unpaired t-test in **F**, **G**, **I**.

**Additional Fig. S2 Hyperphosphorylated tau upregulated reactive oxygen species (ROS) and cytokine level in neuronal cells**

**(A)** Quantification of the immunoreactivity of the blots in figure 2E, normalized against GAPDH. **(B)** ROS levels in SH-SY5Y transfected with vector or TauP301S were quantified by flow cytometry. **(C)** Secretion of TNF-α and IL-6 was quantified using flow cytometry. Data are presented as mean ± standard error of the mean (SEM) of three experiments, and statistical analysis was performed using one-way ANOVA with Dunnett’s multiple comparisons test in **A** and two-tailed unpaired t-test in **B**, **C**.

**Additional Fig. S3 Hyperphosphorylated tau induces necroptosis in HT22 requiring RIPK1, RIPK3 and MLKL**

**(A)** Quantification of the immunoreactivity of the blots in figure 3A, normalized against GAPDH. **(B)** Quantification of the immunoreactivity of the blots in figure 3B, normalized against GAPDH. **(C)** Quantification of the immunoreactivity of the blots in figure 3C, normalized against GAPDH. Data are presented as mean ± standard error of the mean (SEM) of three experiments, and a two-way ANOVA with Sidak's multiple comparisons test was used to analyze the statistical significance of the data.

**Additional Fig. S4 Knockdown of RIPK1, RIPK3 and MLKL inhibits hyperphosphorylated Tau-induced necroptosis**

Representative images of NC, RIPK1-KO, RIPK3-KO and MLKL-KO cells transfected with vector or TauP301S following treatment with DMSO or zVAD (30 μM) or Nec-1 (30 μM) or zVAD (30 μM)+Nec-1 (30 μM) for 24 h, measured using Hoechst 33258/PI staining, Scale bars, 100 μm

**Additional Fig. S5 NF-κB signalling pathway is regulated by the RIPK1-RIPK3-MLKL axis**

**(A)** Quantification of the immunoreactivity of the blots in figure 4A.  **(B)** Quantification of the immunoreactivity of the blots in figure 4K. Data are presented as mean ± standard error of the mean (SEM) of three experiments, and statistical analysis was performed using one-way ANOVA with Dunnett’s multiple comparisons test in **A** and two-way ANOVA with Sidak's multiple comparisons test in **B**

**Additional Fig. S6 Nec-1s treatment reduces neuroinflammation in TauP301S mice**

**(A)** Quantification of the immunoreactivity of the blots in figure 5A, normalized against GAPDH (n=7). **(B)** Quantification of the immunoreactivity of the blots in figure 5D, normalized against GAPDH (n=8-9). **(C)** mRNAs from mice brain were extracted and quantified to determine indicated cytokine levels by qPCR. **(D)** Analysis of pro-inflammatory factors and chemokines in RAB fractions by flow cytometry. **(E)** Quantification of the immunoreactivity of the blots in figure 5E, normalized against GAPDH (n=8-9). **(F)** Quantification of the immunoreactivity of the blots in figure 5F, normalized against GAPDH (n=8-9).Data are presented as mean ± standard error of the mean (SEM) of three experiments, and statistical analysis was performed using two-way ANOVA with Sidak's multiple comparisons test in **A** and one-way ANOVA with Dunnett’s multiple comparisons test in **B**, **C**, **D**, **E**, **F**.

**Additional Table S1. sgRNAs and qPCR primer sequences.**

| gRNA | sequences for CRISPR knock out (5’ – 3’) |
| --- | --- |
| *RIPK1*#1 | TGTGAAAGTCACGATCAACG |
| *RIPK1*#2 | TCCTGGCCACAGGTACAATG |
| *RIPK3*#1 | TGGGGGCACCCTAGCGTACT |
| *RIPK3*#2 | ACCCTCCCTGAAACGTGGAC |
| *MLKL*#1 | CCCAACATCTTGCGTATATT |
| *MLKL*#2 | AGGAACATCTTGGACCTCCG |
| Nontargeting contral | CACCGCTGAAAAAGGAAGGAGTTGA |
| Gene | Primer sequence |
| *IL6* | Forward: 5’-TAGTCCTTCCTACCCCAATTTCC-3’  Reverse: 5’-TTGGTCCTTAGCCACTCCTTC-3’ |
| *TNFα* | Forward: 5’-CATCTTCTCAAAATTCGAGTGACAA-3’  Reverse: 5’-TGGGAGTAGACAAGGTACAACCC-3’ |
| *IFNa4* | Forward: 5’-TGATGAGCTACTACTGGTCAGC-3’  Reverse: 5’-GATCTCTTAGCACAAGGATGGC -3’ |
| *IFNb1* | Forward: 5’-ACTGCCTTTGCCATCCAAGA -3’  Reverse: 5’-AGAAACACTGTCTGCTGGTGG-3’ |
| *Ccl5* | Forward: 5’-CTGCTGCTTTGCCTACCTCT-3’  Reverse: 5’-TCTTCTCTGGGTTGGCACAC-3’ |
| *Cxcl9* | Forward: 5’-GGCATCATCTTCCTGGAGCA-3’  Reverse: 5’-TTGTAGTGGATCGTGCCTCG-3’ |
| *Il15* | Forward: 5’-ACATCCATCTCGTGCTACTTGT-3’ |
|  | Reverse: 5’-GCCTCTGTTTTAGGGAGACCT-3’ |
| *Il1α* | Forward: 5’-CGAAGACTACAGTTCTGCCATT-3’ |
| *TNFSF10* | Reverse: 5’-GACGTTTCAGAGGTTCTCAGAG-3’  Forward: 5’-TATGCCTTCCTCAGGGGC-3  Reverse: 5’-AGGTTCTCAAAGTCACCTCTTCA-3’ |
| *GAPDH* | Forward: 5’-AGGTCGGTGTGAACGGATTTG-3  Reverse: 5’-TGTAGACCATGTAGTTGAGGTCA-3’ |
