# Supplementary figures and images for: Hyperphosphorylated tau mediates neuronal death by inducing necroptosis and inflammation in Alzheimer’s disease
Source: J Neuroinflammation. 2022 Aug 15;19:205. doi: 10.1186/s12974-022-02567-y (PMC9377071; doi:10.1186/s12974-022-02567-y)

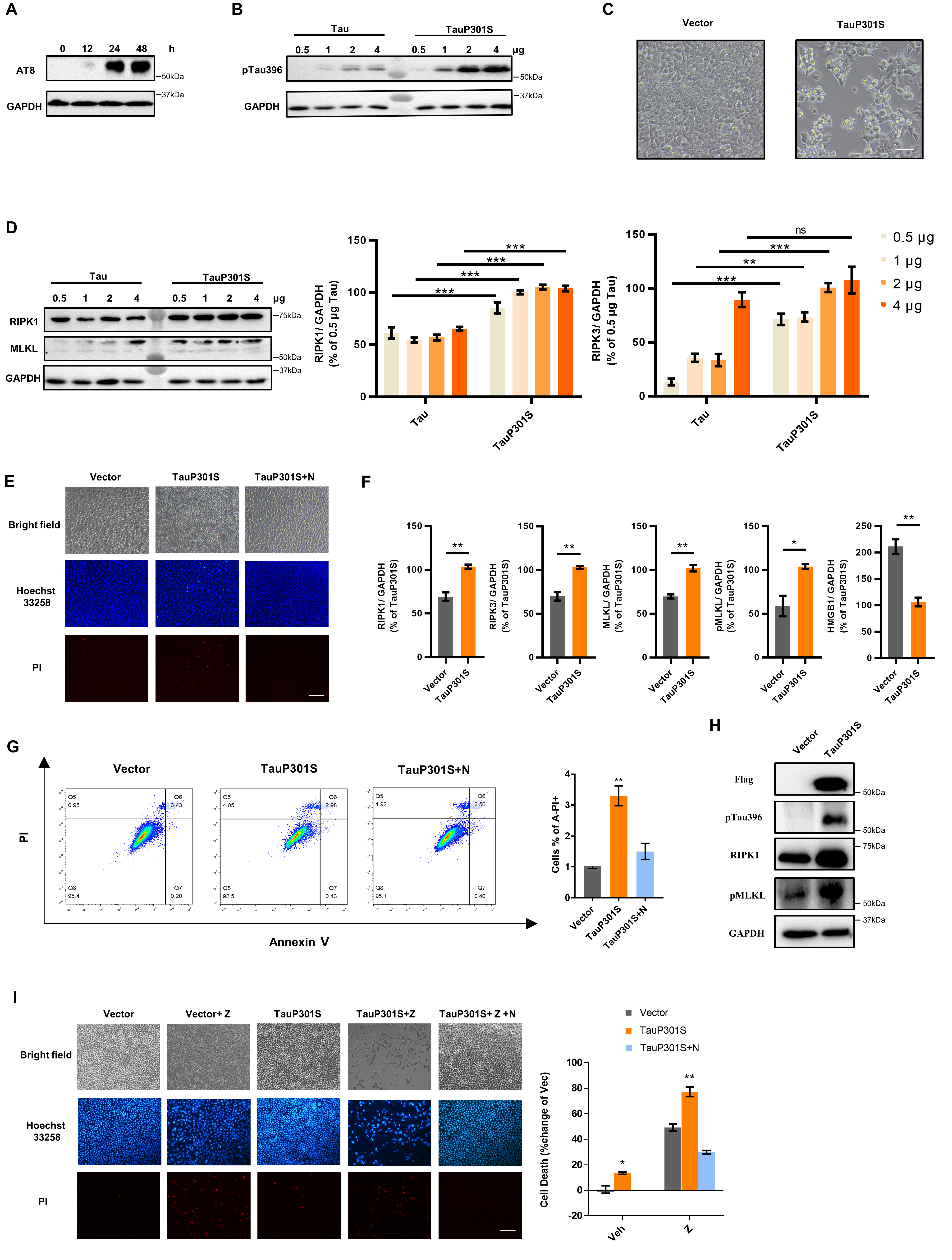

Supplement: Supplementary file 2 — Additional file 2: Figure S1. Necroptosis was stimulated by hyperphosphorylated tau. (A) HEK 293 T cells were transfected with TauP301S for 0, 12, 24, 48 h and the lysates were analyzed by western blotting with AT8. (B) HEK 293 T cells were transfected with 0.5, 1, 2 or 4 μg TauP301S and the lysates were analyzed by western blotting using indicated antibodies. (C) Representative images of HEK 293 T cells transfected with vector or TauP301S in bright field, Scale bars, 50 μm. (D) HEK 293 T cells were transfected with 0.5, 1, 2 or 4 μg TauP301S and the lysates were analyzed by western blotting using indicated antibodies, quantification of the immunoreactivity of the blots, normalized against GAPDH (E) Representative images of HT22 cells transfected with vector or TauP301S, followed by treatment with DMSO or Nec-1 (30 μM) for 24 h and examined by Hoechst 33258/PI staining, Scale bars, 100 μm. (F) Quantification of the immunoreactivity of the blots in Fig. 1D, normalized against GAPDH. (G) SH-SY5Y cells were transfected with vector or TauP301S, followed by treatment with DMSO or Nec-1 (30 μM) for 48 h; cell death was analyzed by flow cytometry using Annexin V/PI staining. (H) SH-SY5Y cells were transfected with vector or TauP301S, and the lysates were analyzed by western blotting using indicated antibodies. (I) Representative images of HT22 cells transfected with vector or TauP301S, followed by treatment with DMSO or zVAD (30 μM) or zVAD (30 μM) + Nec-1 (30 μM) for 24 h and examined by Hoechst 33258/PI staining, Scale bars, 10 μm; cell death was quantified by measuring LDH levels. Data are presented as the mean ± standard error of the mean (SEM) of three experiments, and statistical analysis was performed using two-way ANOVA with Tukey’s multiple comparisons test in D and two-tailed unpaired t test in F, G, I. [file 12974_2022_2567_MOESM2_ESM.tif]

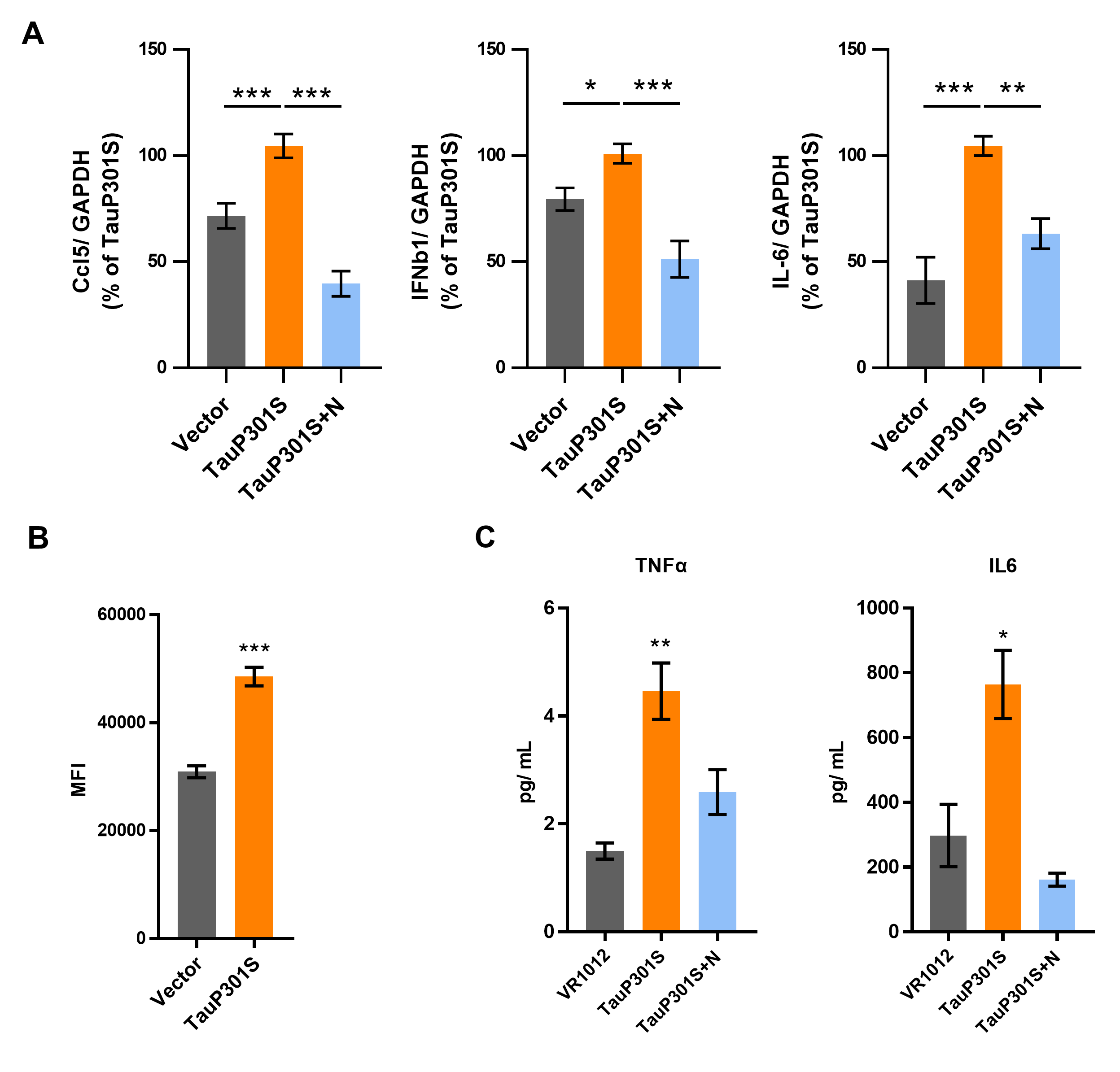

Supplement: Supplementary file 3 — Additional file 3: Figure S2. Hyperphosphorylated tau upregulated reactive oxygen species (ROS) and cytokine level in neuronal cells. (A) Quantification of the immunoreactivity of the blots in Fig. 2E, normalized against GAPDH. (B) ROS levels in SH-SY5Y transfected with vector or TauP301S were quantified by flow cytometry. (C) Secretion of TNF-α and IL-6 was quantified using flow cytometry. Data are presented as mean ± standard error of the mean (SEM) of three experiments, and statistical analysis was performed using one-way ANOVA with Dunnett’s multiple comparisons test in A and two-tailed unpaired t test in B, C. [file 12974_2022_2567_MOESM3_ESM.tif]

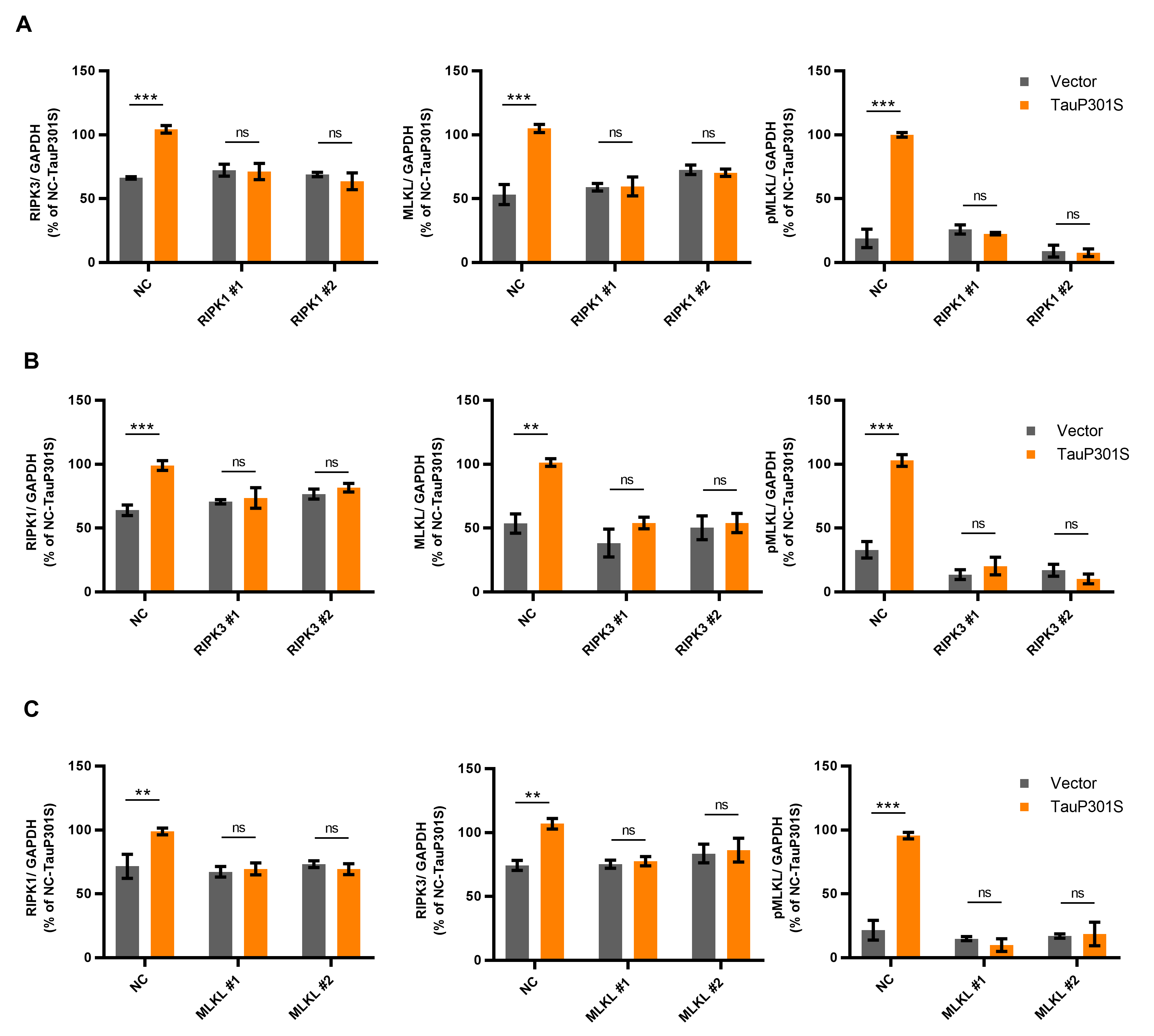

Supplement: Supplementary file 4 — Additional file 4: Figure S3. Hyperphosphorylated tau induces necroptosis in HT22 requiring RIPK1, RIPK3 and MLKL. (A) Quantification of the immunoreactivity of the blots in Fig. 3A, normalized against GAPDH. (B) Quantification of the immunoreactivity of the blots in Fig. 3B, normalized against GAPDH. (C) Quantification of the immunoreactivity of the blots in Fig. 3C, normalized against GAPDH. Data are presented as mean ± standard error of the mean (SEM) of three experiments, and a two-way ANOVA with Sidak's multiple comparisons test was used to analyze the statistical significance of the data. [file 12974_2022_2567_MOESM4_ESM.tif]

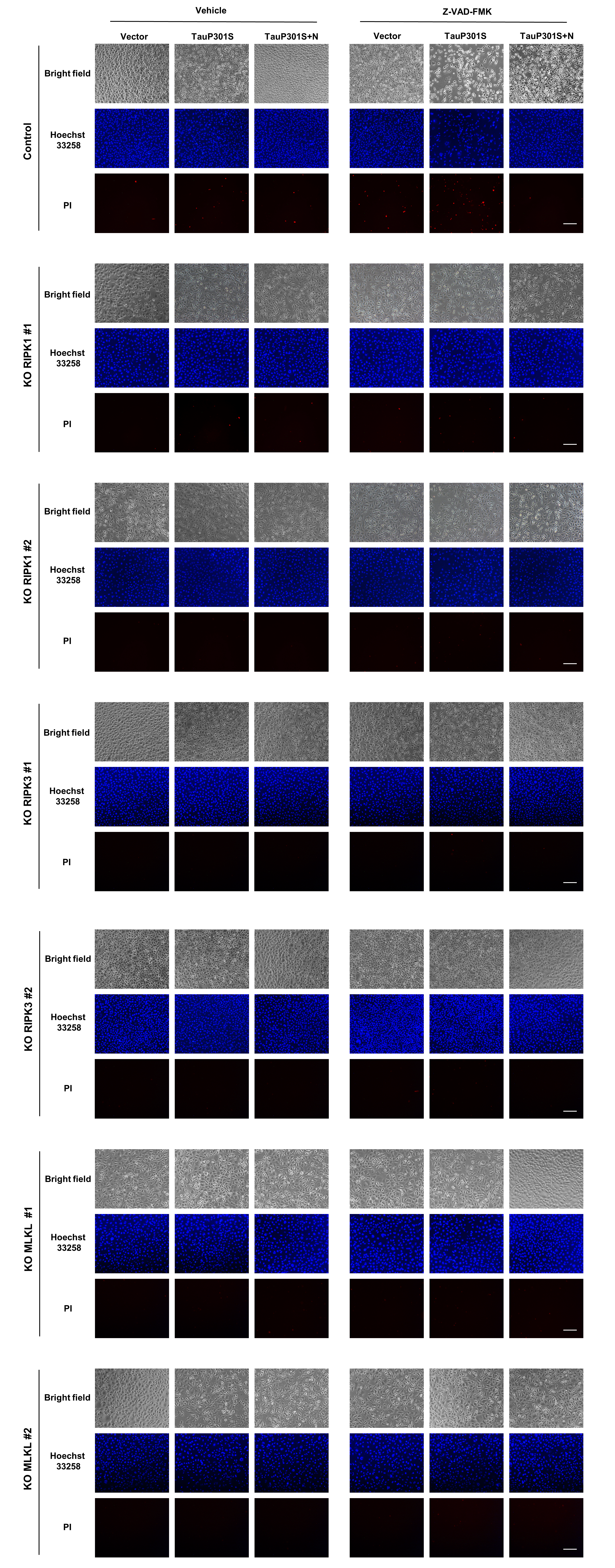

Supplement: Supplementary file 5 — Additional file 5: Figure S4. Knockdown of RIPK1, RIPK3 and MLKL inhibits hyperphosphorylated Tau-induced necroptosis. Representative images of NC, RIPK1-KO, RIPK3-KO and MLKL-KO cells transfected with vector or TauP301S following treatment with DMSO or zVAD (30 μM) or Nec-1 (30 μM) or zVAD (30 μM) + Nec-1 (30 μM) for 24 h, measured using Hoechst 33258/PI staining, Scale bars, 100 μm. [file 12974_2022_2567_MOESM5_ESM.tif]

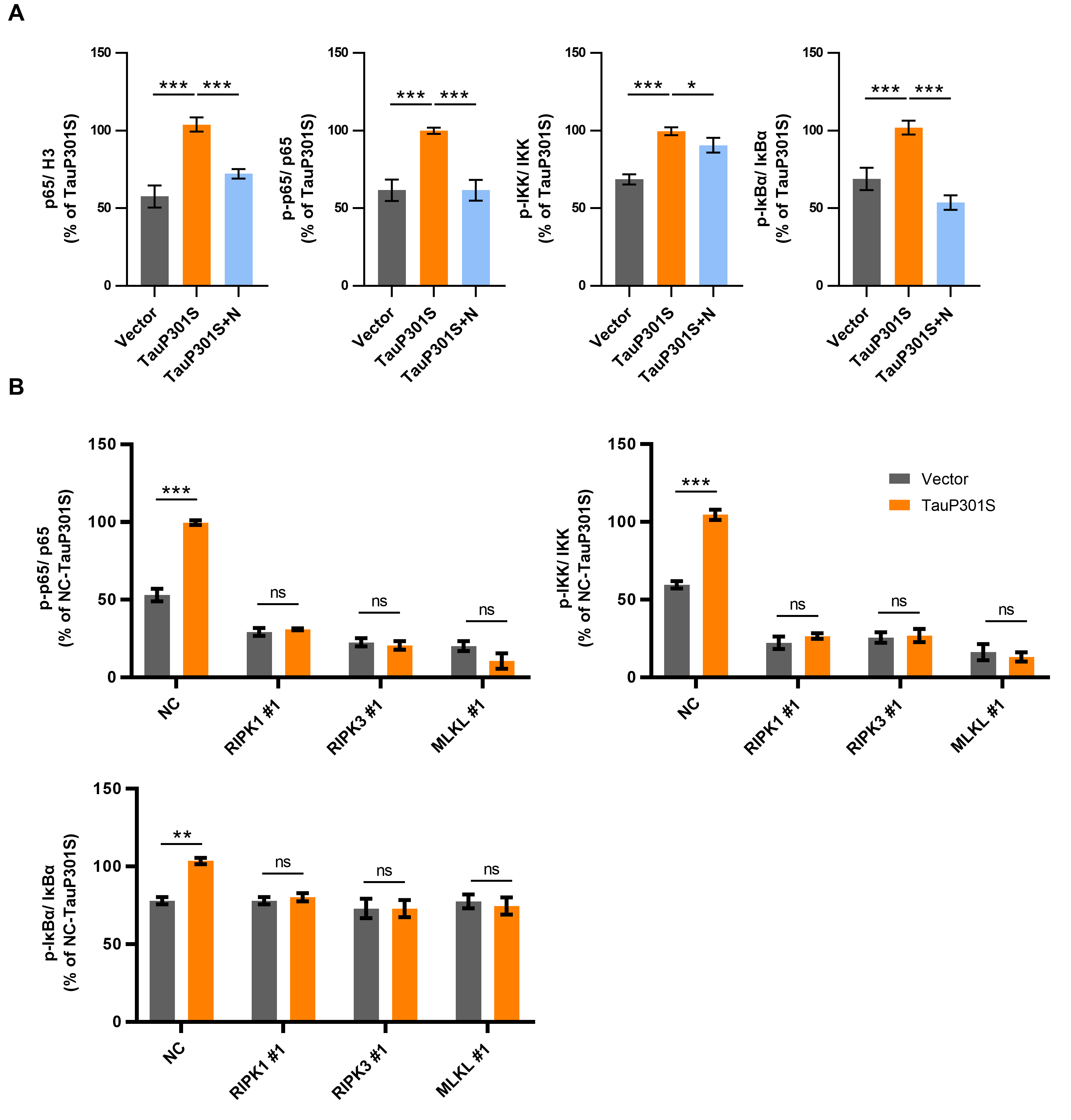

Supplement: Supplementary file 6 — Additional file 6: Figure S5. NF-κB signalling pathway is regulated by the RIPK1–RIPK3–MLKL axis. (A) Quantification of the immunoreactivity of the blots in Fig. 4A. (B) Quantification of the immunoreactivity of the blots in Fig. 4K. Data are presented as mean ± standard error of the mean (SEM) of three experiments, and statistical analysis was performed using one-way ANOVA with Dunnett’s multiple comparisons test in A and two-way ANOVA with Sidak's multiple comparisons test in B. [file 12974_2022_2567_MOESM6_ESM.tif]

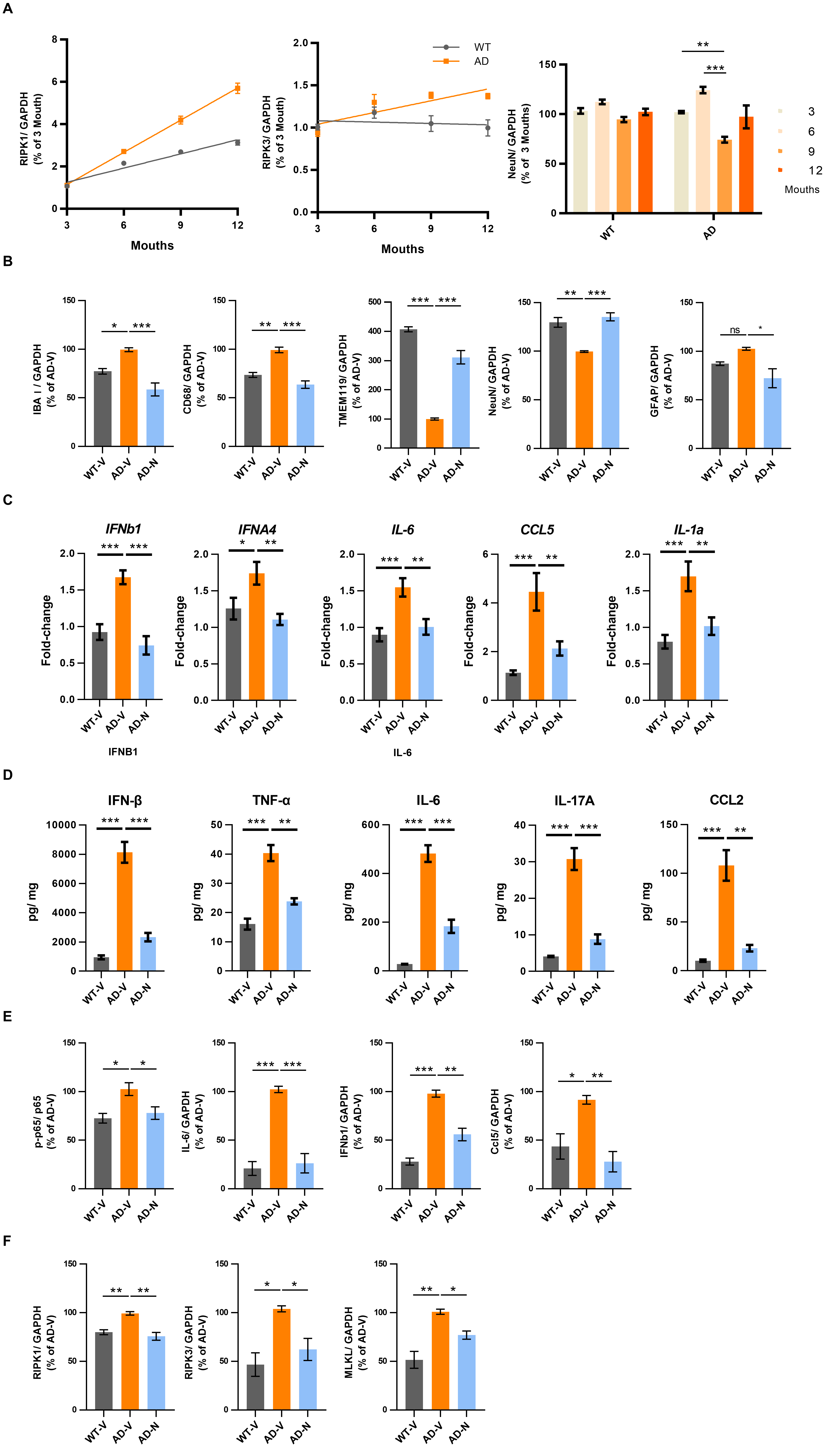

Supplement: Supplementary file 7 — Additional file 7: Figure S6. Nec-1 s treatment reduces neuroinflammation in TauP301S mice. (A) Quantification of the immunoreactivity of the blots in Fig. 5A, normalized against GAPDH (n = 7). (B) Quantification of the immunoreactivity of the blots in Fig. 5D, normalized against GAPDH (n = 8–9). (C) mRNAs from mice brain were extracted and quantified to determine indicated cytokine levels by qPCR. (D) Analysis of pro-inflammatory factors and chemokines in RAB fractions by flow cytometry. (E) Quantification of the immunoreactivity of the blots in Fig. 5E, normalized against GAPDH (n = 8–9). (F) Quantification of the immunoreactivity of the blots in Fig. 5F, normalized against GAPDH (n = 8–9).Data are presented as mean ± standard error of the mean (SEM) of three experiments, and statistical analysis was performed using two-way ANOVA with Sidak's multiple comparisons test in A and one-way ANOVA with Dunnett’s multiple comparisons test in B, C, D, E, F. [file 12974_2022_2567_MOESM7_ESM.tif]

Figure 1

D

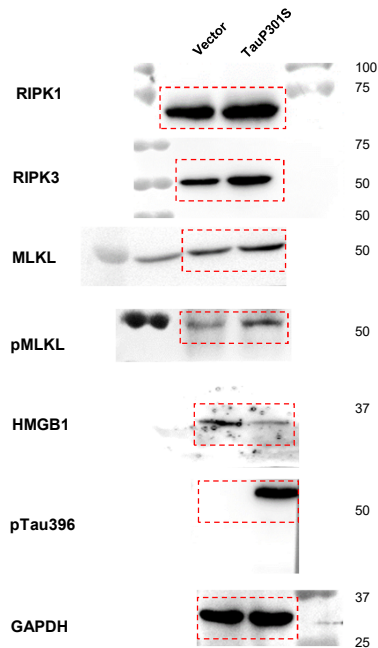

E

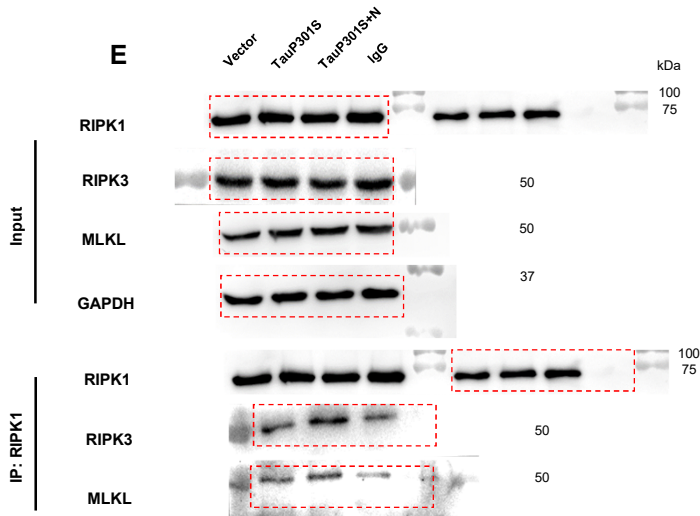

F

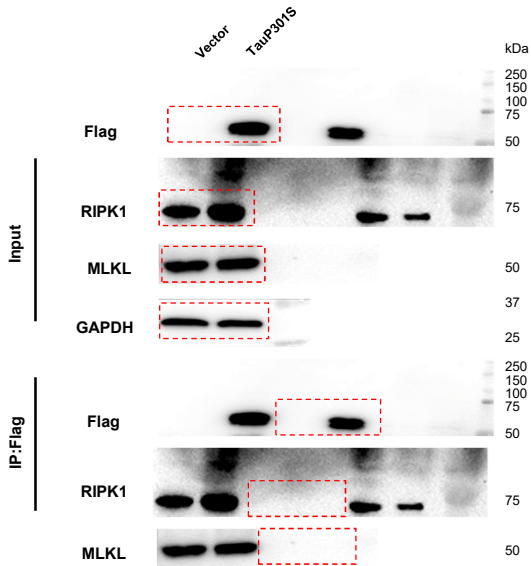

Figure 2

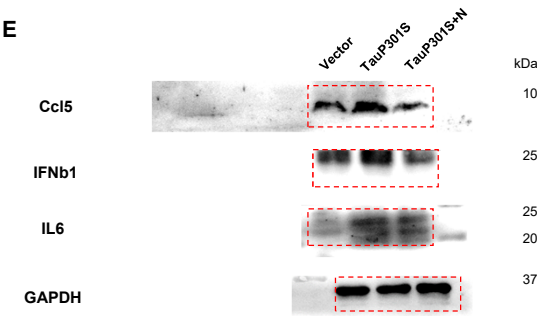

Figure 3

A

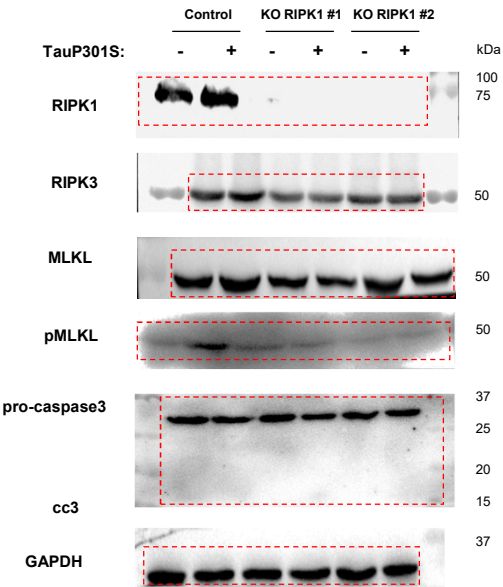

B

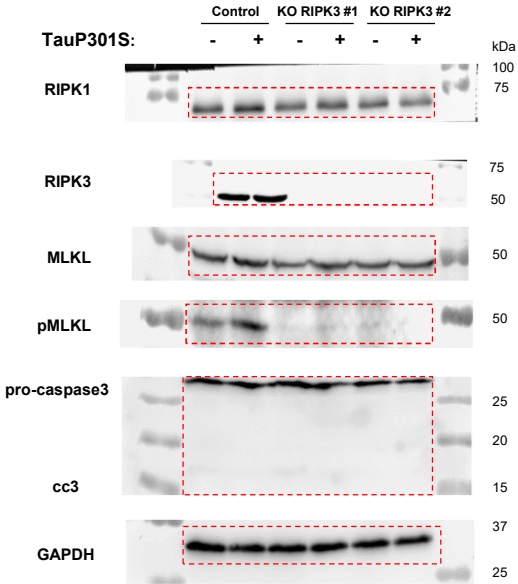

C

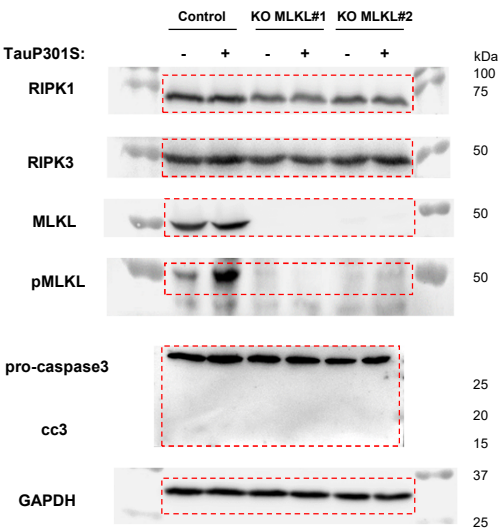

Figure 4

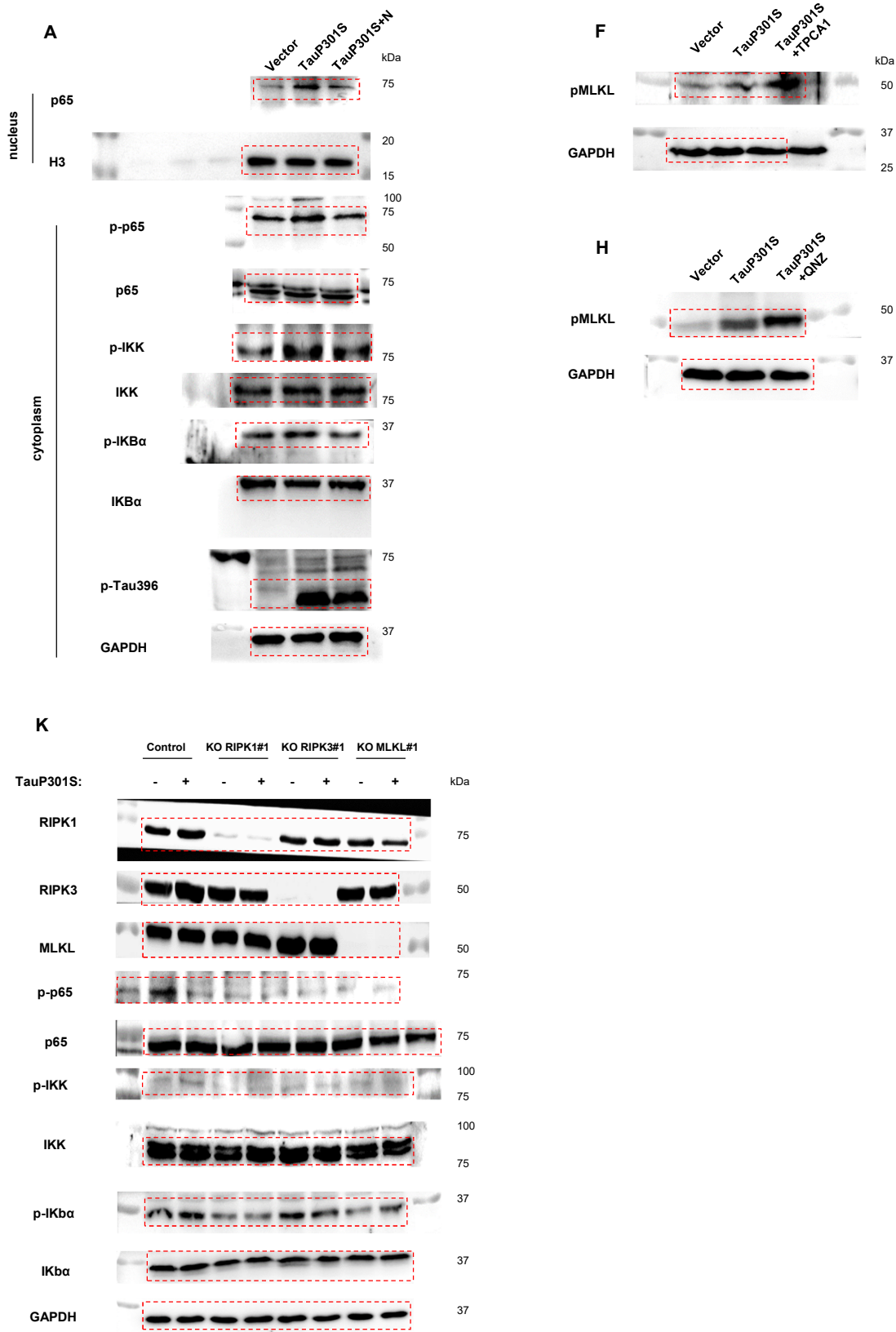

Figure 5

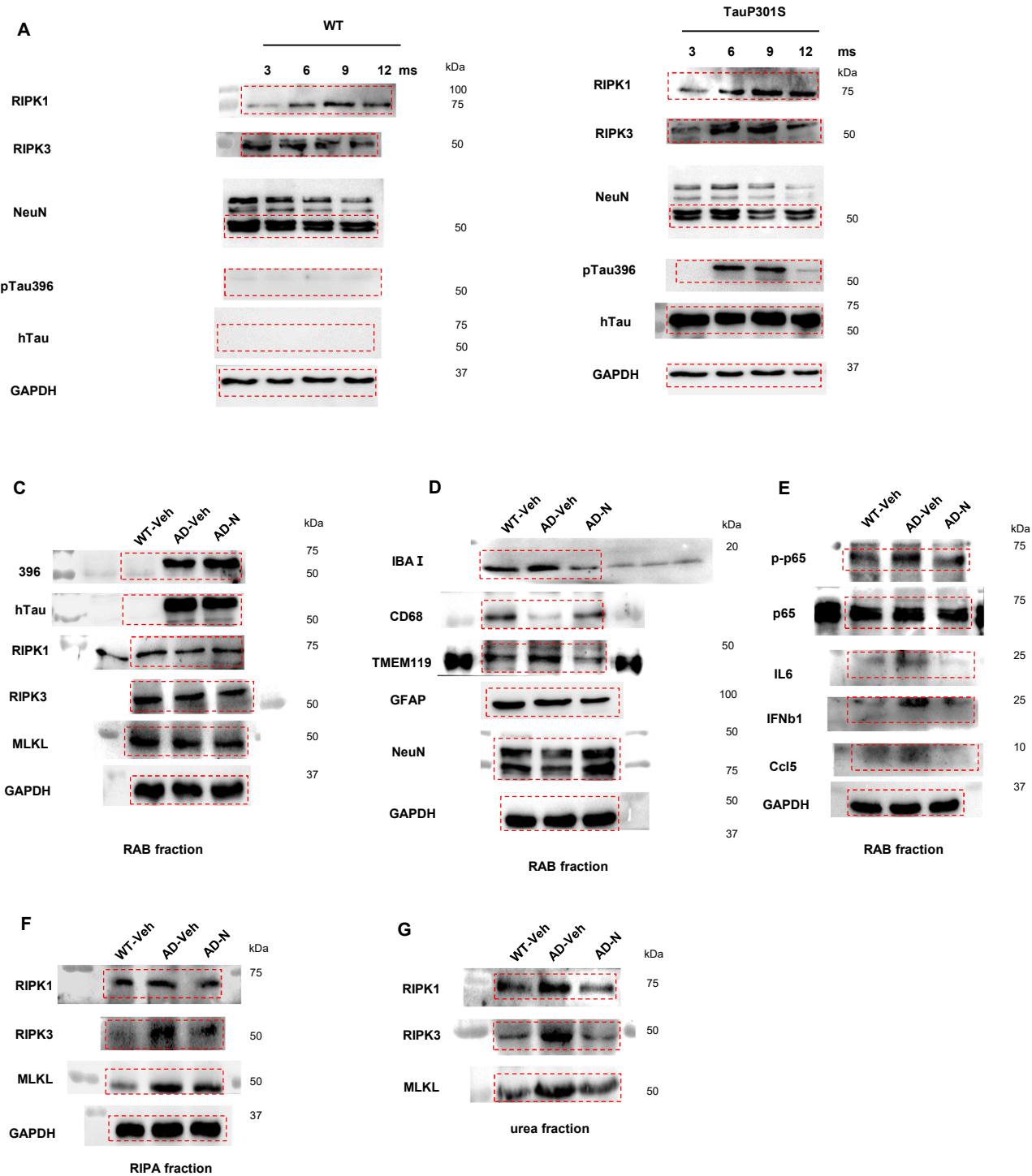

Figure S1

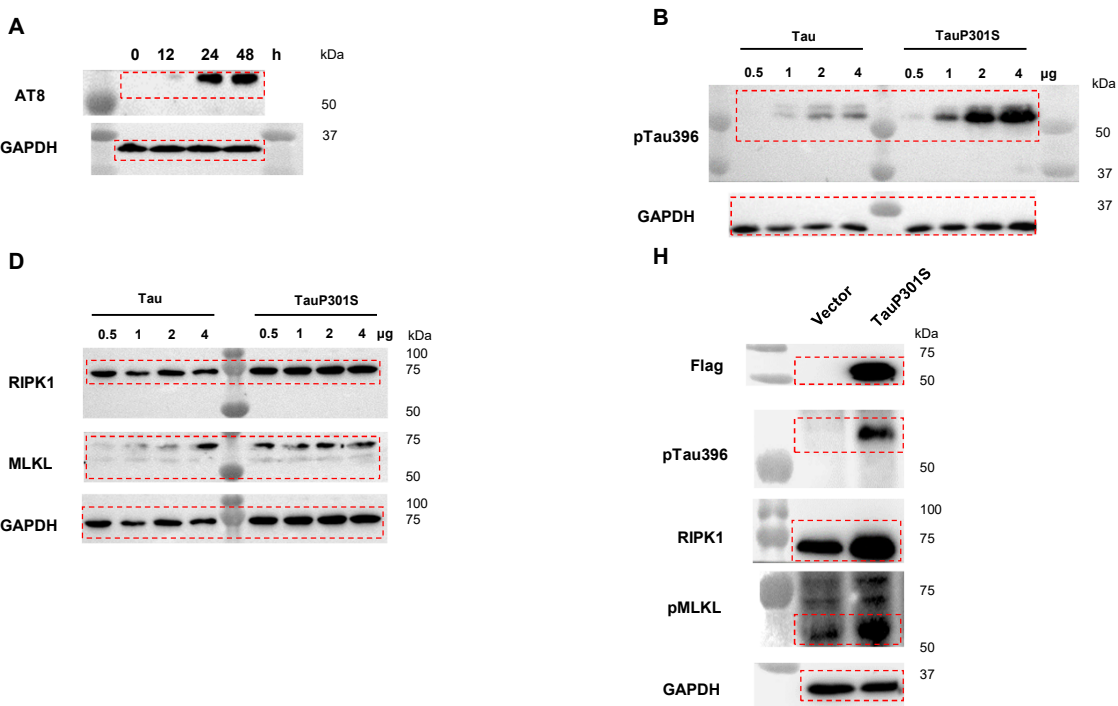

Supplement: Supplementary file 9 — Additional file 9. Raw images of Western blot. [file 12974_2022_2567_MOESM9_ESM.pdf]
